# Supplementary material for: Multiple protein-DNA interfaces unravelled by evolutionary information, physico-chemical and geometrical properties
Source: PLoS Comput Biol. 2020 Feb 3;16(2):e1007624. doi: 10.1371/journal.pcbi.1007624 (PMC7018136; doi:10.1371/journal.pcbi.1007624)
Supplement: S1 File — (PDF) [file pcbi.1007624.s001.pdf]

Supplementary Tables

References

[1] H. M. Berman, J. Westbrook, Z. Feng, G. Gilliland, T. N. Bhat, H. Weissig, I. N. Shindyalov, and P. E. Bourne, "The protein data bank," *Nucleic Acids Research*, vol. 28, no. 1, pp. 235–242, 2000.

[2] H. M. Berman, W. K. Olson, D. L. Beveridge, J. Westbrook, A. Gelbin, T. Demeny, S.-H. Hsieh, A. Srinivasan, and B. Schneider, "The nucleic acid database. a comprehensive relational database of three-dimensional structures of nucleic acids," *Biophysical Journal*, vol. 63, no. 3, pp. 751–759, 1992.

[3] J. Yan and L. Kurgan, "Drnapred, fast sequence-based method that accurately predicts and discriminates dna-and rna-binding residues," *Nucleic acids research*, vol. 45, no. 10, pp. e84–e84, 2017.

[4] S. Selvaraj, H. Kono, and A. Sarai, "Specificity of protein–dna recognition revealed by structure-based potentials: symmetric/asymmetric and cognate/non-cognate binding," *Journal of Molecular Biology*, vol. 322, no. 5, pp. 907–915, 2002.

[5] S. Ahmad, M. M. Gromiha, and A. Sarai, "Analysis and prediction of dna-binding proteins and their binding residues based on composition, sequence and structural information," *Bioinformatics*, vol. 20, no. 4, pp. 477–486, 2004.

[6] J. Wu, H. Liu, X. Duan, Y. Ding, H. Wu, Y. Bai, and X. Sun, "Prediction of dna-binding residues in proteins from amino acid sequences using a random forest model with a hybrid feature," *Bioinformatics*, vol. 25, no. 1, pp. 30–35, 2008.

[7] J. Si, Z. Zhang, B. Lin, M. Schroeder, and B. Huang, "Metadbsite: a meta approach to improve protein dna-binding sites prediction," *BMC Systems Biology*, vol. 5, no. 1, p. S7, 2011.

[8] H. Tjong and H.-X. Zhou, "Displar: an accurate method for predicting dna-binding sites on protein surfaces," *Nucleic Acids Research*, vol. 35, no. 5, pp. 1465–1477, 2007.

[9] M. Van Dijk and A. M. Bonvin, "A protein–dna docking benchmark," *Nucleic Acids Research*, vol. 36, no. 14, pp. e88–e88, 2008.

[10] Y. Xiong, J. Liu, and D.-Q. Wei, "An accurate feature-based method for identifying dna-binding residues on protein surfaces," *Proteins: Structure, Function, and Bioinformatics*, vol. 79, no. 2, pp. 509–517, 2011.

[11] T. Li, Q.-Z. Li, S. Liu, G.-L. Fan, Y.-C. Zuo, and Y. Peng, "Predna: accurate prediction of dna-binding sites in proteins by integrating sequence and geometric structure information," *Bioinformatics*, vol. 29, no. 6, pp. 678–685, 2013.

[12] P. Ozbek, S. Soner, B. Erman, and T. Haliloglu, "Dnabindprot: fluctuation-based predictor of dna-binding residues within a network of interacting residues," *Nucleic Acids Research*, vol. 38, no. suppl_2, pp. W417–W423, 2010.

Table A: **List of the 187 complexes comprised in HR-PDNA187 dataset and the 82 HOLO-APO pairs available.** The entries in the columns are respectively: 1) the Protein Data Bank (PDB) [1] identifier of the HR-PDNA187 complex; 2) the protein chains considered in the complex; 3) the subset of protein chains reported in column 2 non redundant at 95% of sequence identity; 4) the DNA chains considered in the complex; 5) the PDB identifier and the considered chains of the HOLO form in the HOLO-APO82 dataset; 6) the PDB identifier and the considered chains of the APO form in the HOLO-APO82 dataset; 7) the class and 8) the subclass to which the protein belongs as derived from the Nucleic Acid Database [2]. Concerning the protein stoichiometry, the HR-PDNA187 comprises: 109 monomers, 64 homo-2-mers, 3 hetero-2-mers, 3 homo-3-mers, 1 hetero-3-mer, 5 homo-4-mers, 1 hetero-5-mer and 1 homo-6-mer. The APO forms in the HOLO-APO82 dataset are divided in: 52 monomers, 28 homo-2-mers, 1 homo-3-mer and 1 hetero-3-mer. Specifically, we observed a change in the stoichiometry in 4 HOLO-APO pairs: 3 homo-4-mers, 1 homo-4-mer, 1 homo-3-mer, 1 homo-2-mer in the bound form are respectively 3 homo-2-mers and 3 monomers in the unbound form.

PDB ID	prot chains	prot nr chains	DNA chains	HOLO ID:c	APO ID:c	Class	Subclass
1a3q	AB	A	CD			regulatory	transcription factor
1a73	AB	A	CDEF	1a73:BA	1evx:AB	enzyme	nuclease
1b3t	AB	A	CD	1b3t:BA	1vhi:AB	regulatory	other
1bdt	ABCD	A	EF	1bdt:CD	1myk:AB	regulatory	gene
1bl0	A	A	BC			regulatory	transcription factor
1cez	A	A	NT			enzyme	polymerase
1d02	AB	A	CD			enzyme	nuclease
1dc1	AB	A	CW			enzyme	nuclease
1dfm	AB	A	CD			enzyme	nuclease
1egw	AB	A	EF			regulatory	transcription factor
1emh	A	A	BC	1emh:A	3fci:A	enzyme	glycosylase
1esg	AB	A	CD			enzyme	nuclease
1f4k	AB	A	DE	1f4k:BA	2dqr:AB	regulatory	replication
1fiu	ABCD	A	EFGHIJKL			enzyme	nuclease
1gu4	AB	A	CD			regulatory	transcription factor
1gxp	AB	A	CD	1gxp:B	1gxq:A	regulatory	other
1h6f	AB	A	CD			regulatory	transcription factor
1hlv	A	A	BC			structural	other/centromere
1i3j	A	A	BC			enzyme	nuclease
1iaw	AB	A	CDEF	1iaw:BA	1ev7:AB	enzyme	hydrolase
1j3e	A	A	BC			regulatory	replication
1je8	AB	A	CD	1je8:B	1a04:A	regulatory	transcription factor
1jko	C	C	AB			enzyme	recombinase
1jx4	A	A	PT	1jx4:A	2rdi:A	enzyme	polymerase
1k3x	A	A	BC	1k3x:A	1q39:A	enzyme	nuclease
1k4t	A	A	BCD			enzyme	isomerase
1ku7	A	A	BC	1ku7:A	1ku3:A	regulatory	transcription factor
1kx5	ABCDEFGH	B<br>D<br>A<br>C	IJ			structural	histone
1l3l	BD	B	EG			regulatory	transcription factor
1lmb	34	3	12			regulatory	other
1lq1	CD	C	EF			regulatory	transcription factor
1mjo	ABCD	A	FG	1mjo:AB	1mjl:AB	regulatory	transcription factor
1mnn	A	A	BC	1mnn:A	1mn4:A	regulatory	transcription factor
1nkp	AB	B<br>A	FG			regulatory	transcription factor
1oe4	AB	A	EF			enzyme	glycosylase
1orn	A	A	BC			enzyme	nuclease
1oup	B	B	CD	1oup:B	1ouo:A	enzyme	nuclease
1owf	AB	A<br>B	CDE			regulatory	transcription factor
1ozj	A	A	CD			regulatory	transcription factor

Continued on next page

Table A – continued from previous page

PDB ID	prot chains	prot nr chains	DNA chains	HOLO ID:c	APO ID:c	Class	Subclass
1pp7	U	U	EF			regulatory	transcription factor
1pt3	A	A	CDEFGH	1pt3:A	3zfk:A	enzyme	nuclease
1qna	A	A	CD	1qna:A	1vok:A	regulatory	transcription factor
1r71	AB	A	EFIJ			regulatory	transcription factor
1rh6	B	B	CD			regulatory	recombination
1rxw	A	A	BC			enzyme	nuclease
1sa3	A	A	CD			enzyme	nuclease
1skn	P	P	AB			regulatory	transcription factor
1sx5	AB	A	CDEF	1sx5:AB	1az3:AB	enzyme	nuclease
1sxq	A	A	CE	1sxq:A	1jg7:A	enzyme	transferase
1t7p	A	A	PT			enzyme	polymerase
1t9i	AB	A	CD	1t9i:AB	2o7m:AB	enzyme	nuclease
1tc3	C	C	AB			enzyme	other
1tez	A	A	IJK	1tez:A	1owl:A	enzyme	lyase
1u8b	A	A	BCDE			regulatory	other
1uut	A	A	C	1uut:A	1m55:A	enzyme	nuclease
1wb9	AB	A	EF			regulatory	repair
1xyi	A	A	BC			structural	chromosomal
1yf3	A	A	CD	1yf3:A	1q0s:A	enzyme	methyl
1yo5	C	C	AB			regulatory	transcription factor
1zme	CD	C	AB			regulatory	transcription factor
1zrf	AB	A	WXYZ	1zrf:AB	4r8h:AB	regulatory	other
2aor	A	A	CD			enzyme	methyl
2aq4	A	A	PT			enzyme	transferase
2bnw	ABCD	A	EFGH	2bnw:CD	1irq:AB	regulatory	other
2dp6	A	A	CD	2dp6:A	2d3y:A	enzyme	glycosylase
2e52	AB	A	EG			enzyme	nuclease
2ex5	AB	A	XY			enzyme	nuclease
2fkc	A	A	CD	2fkc:A	1ynm:A	enzyme	nuclease
2g1p	A	A	FG	2g1p:A	4gom:D	enzyme	methyl
2gb7	AB	A	EF			enzyme	nuclease
2h27	A	A	BC			enzyme	transferase
2h7g	X	X	YZ			enzyme	isomerase
2i06	A	A	BC			regulatory	replication
2ih2	A	A	BC	2ih2:A	1aqj:B	enzyme	methyl
2ihm	A	A	DPT			enzyme	polymerase
2is6	A	A	CD	2is6:A	3lfu:A	enzyme	helicase
2isz	AB	A	EF	2isz:BA<br>2isz:B	2isy:AB<br>1b1b:A	regulatory	transcription factor
2noh	A	A	BC	2noh:A	5an4:A	enzyme	glycosylase
2nq9	A	A	BCD	2nq9:A	1qtw:A	enzyme	nuclease
2o4a	A	A	BC			regulatory	transcription factor
2ofi	A	A	BC	2ofi:A	2ofk:A	enzyme	glycosylase
2pi0	B	B	EF	2pi0:B	3qu6:A	regulatory	other
2pyj	A	A	XY	2pyj:A	1xhx:A	enzyme	polymerase
2qhb	B	B	CD	2qhb:B	2ckx:A	structural	telomere
2qoj	Z	Z	XY			enzyme	nuclease
2r1j	LR	L	AB			regulatory	transcription factor
2r9l	A	A	CD	2r9l:A	2iru:A	enzyme	polymerase
2rbf	AB	A	CD	2rbf:BA	2gpe:AB	regulatory	other
2ve9	ABC	A	IJ	2ve9:A	2ve8:A	structural	other
2vla	A	A	LM			enzyme	nuclease
2vs7	A	A	BC	2vs7:A	1b24:A	enzyme	nuclease
2w42	A	A	PQ	2w42:A	1w9h:A	regulatory	other
2w7n	AB	A	EFGH	2w7n:BA	5ckt:AD	regulatory	gene
2xm3	CD	C	KLMN			enzyme	other/transposase
2xrz	A	A	CD	2xrz:A	2xry:A	enzyme	lyase
2xzf	A	A	BC			enzyme	glycosylase

Continued on next page

Table A – continued from previous page

PDB ID	prot chains	prot nr chains	DNA chains	HOLO ID:c	APO ID:c	Class	Subclass
2yvh	AB	A	EFGH	2yvh:AB	2yve:AB	regulatory	transcription factor
3aaf	A	A	CD			enzyme	other
3bep	AB	A	CD	3bep:BA	4k3l:AB	enzyme	polymerase
3bm3	AB	A	CD			enzyme	nuclease
3bs1	A	A	BC	3bs1:A	4g4k:A	regulatory	gene
3c0w	A	A	BCD			enzyme	nuclease
3c25	AB	A	CD	3c25:AB	3bvq:AB	enzyme	nuclease
3coq	AB	A	DE			regulatory	transcription factor
3cw7	ABCD	A	EFGH	3cw7:B	1mpg:A	enzyme	glycosylase
3dsd	AB	A	C	3dsd:BA	1ii7:AB	regulatory	repair
3dvo	AB	A	EF			enzyme	nuclease
3eeo	A	A	CD	3eeo:A	1hmy:A	enzyme	methyl
3f2b	A	A	PT			enzyme	polymerase
3fde	A	A	DE	3fde:A	2zkg:A	enzyme	ligase
3fdq	AB	A	CD			regulatory	other
3g00	A	A	HI	3g00:A	3g8v:A	enzyme	nuclease
3g0q	A	A	BC			enzyme	hydrolase
3g9m	AB	A	CD			regulatory	transcription factor
3gox	AB	A	CD			enzyme	nuclease
3gxq	AB	A	CD			regulatory	other
3h0d	AB	A	CD			regulatory	transcription factor
3i0w	A	A	BC	3i0w:A	3f10:A	enzyme	glycosylase
3iag	C	C	AB			regulatory	transcription factor
3iay	A	A	PT			enzyme	polymerase
3igm	AB	A	CDWX			regulatory	transcription factor
3ikt	AB	A	CD	3ikt:AB	3ikv:AB	regulatory	other
3jso	AB	A	CD	3jso:AB	1jhf:AB	regulatory	other
3jxy	A	A	BC	3jxy:A	3bvs:A	enzyme	glycosylase
3k59	A	A	PT	3k59:A	3k5o:A	enzyme	polymerase
3kde	C	C	AB			enzyme	other
3kxt	A	A	BC			structural	other
3l2c	A	A	BC			regulatory	transcription factor
3lap	ABCDEF	A	GHIJKL			regulatory	other
3m4a	A	A	DE	3m4a:A	2f4q:A	enzyme	isomerase
3mfi	A	A	PT	3mfi:A	1jih:A	enzyme	polymerase
3mln	AB	A	CD			regulatory	transcription factor
3mva	O	O	DE			regulatory	transcription factor
3mx4	AH	A	KL			enzyme	nuclease
3o1t	A	A	BC	3o1t:A	4jht:A	enzyme	other
3o9x	AB	A	EF	3o9x:AB	3gn5:AB	regulatory	gene
3od8	A	A	IJ			enzyme	other
3pov	A	A	CD	3pov:A	3fhd:A	enzyme	other
3pvi	AB	A	CD	3pvi:AB	1k0z:AB	enzyme	nuclease
3pvv	A	A	CD			regulatory	replication
3qex	A	A	PT	3qex:A	3cfo:A	enzyme	polymerase
3qmd	A	A	BC			regulatory	other
3qqy	A	A	BC			enzyme	nuclease
3qws	AB	A	CN	3qws:AB	2hin:AB	regulatory	other
3rkq	A	A	CD			regulatory	transcription factor
3rmp	AC	A	EFGH			enzyme	other
3s57	A	A	BC			enzyme	other
3s8q	AB	A	CD	3s8q:BA	4i6r:AB	regulatory	other
3sjm	A	A	CD			structural	telomere
3sm4	ABC	A	DE	3sm4:CAB	1avq:ABC	enzyme	nuclease
3spd	A	A	EF	3spd:A	3sp4:A	enzyme	hydrolase
3ssc	A	A	CD			enzyme	nuclease
3tan	A	A	BC			enzyme	polymerase
3tq6	A	A	CD			regulatory	transcription factor

Continued on next page

Table A – continued from previous page

PDB ID	prot chains	prot nr chains	DNA chains	HOLO ID:c	APO ID:c	Class	Subclass
3u2b	C	C	AB			regulatory	transcription factor
3vk8	A	A	CD	3vk8:A	3a42:A	enzyme	glycosylase
3vxv	A	A	BC			enzyme	hydrolase
3zvk	FG	E	XY			regulatory	other
3zvn	A	A	EFGHI	3zvn:A	3zvl:A	enzyme	hydrolase
4aij	AB	A	CD	4aij:BA	4aih:AB	regulatory	transcription factor
4dih	H	H	D	4dih:H	3nxp:A	enzyme	thrombin
4e9f	A	A	CD	4e9f:A	4e9e:A	enzyme	Hydrolase/glycosylase
4ecq	A	A	PT			enzyme	polymerase
4esj	A	A	CD			enzyme	nuclease
4fzx	C	C	AB			enzyme	nuclease
4g92	ABC	B<br>C<br>A	DE	4g92:ABC	4g91:ABC	regulatory	transcription factor
4gck	AB	A	WZ	4gck:AB	4gfl:AB	other	other
4gjr	AB	A	GHIJ			regulatory	transcription factor
4glx	A	A	BCD	4glx:A	5tt5:A	enzyme	ligase
4gzn	C	C	AB			regulatory	transcription factor
4h0e	B	B	TU			regulatory	transcription factor
4h10	AB	B<br>A	CD			regulatory	transcription factor
4hf1	AB	A	CD	4hf1:AB	4hf0:AB	regulatory	transcription factor
4hqe	AB	A	CD	4hqe:BA	4hqm:AB	regulatory	transcription factor
4htu	A	A	CD	4htu:A	1zbf:A	enzyme	nuclease
4i2o	AB	A	XW			regulatory	other
4ix7	AB	A	CD			regulatory	other
4j3n	AB	A	CDEF			enzyme	isomerase
4jbm	A	A	RT			regulatory	other
4jcy	AB	A	CD	4jcy:BA	3lis:AB	regulatory	other
4k98	A	A	DE	4k98:A	4k8v:C	enzyme	transferase
4kb1	A	A	C			enzyme	hydrolase
4kli	A	A	DPT			enzyme	polymerase
4kpy	A	A	CDN			NoClass	NoClass
4qtj	A	A	BC			regulatory	transcription factor
4rkh	CEF	C	AB			enzyme	ligase
6pax	A	A	BC			regulatory	transcription factor

Table B: **List of 24 complexes present in TEST-DBP24, annotated with the reasons they were excluded from HR-PDNA187.**

3UGO, 4FPV, 4GZ0, 4HIO, 4HQB, 4JRP, 4LJR, 4LUP, 4OWX	discarded in our dataset because single stranded DNA
3VOK, 3ZDB, 4E0J, 4HLY, 4HQX	discarded in our dataset because the asymmetric unit does not contain a complete biological unit
3ZH2, 4BXO, 4LMG, 4NDH, 4NI7, 4NM6	not present in our original list downloaded from NDB (Nucleic Acids Database)
3VD6	homologous to 4HC9 and thus discarded in our dataset by PISCES; then 4HC9 was itself manually discarded in our dataset because interfaces of asymmetric and biological units did not match
3U3W, 4HP1, 4L0Y	discarded in our dataset by PISCES because having a sequence identity in the range 25-30% compared to 3S8Q, 3QMD, 1YO5, respectively.

Table C: $\mathbf{JET^2_{DNA}}$ **algorithm**

Seeds detection

```
R ← {r_i, score(r_i) > score_res^seed};
for r_i ∈ R and ∉ C do
    if score(r_i) > score_clus^seed then
        newClus ← r_i;
        while {neighbors of newClus}_∈R ≠ {} do
            for r_j ∈ {neighbors of newClus}_∈R do
                if μ(newClus + r_j) > score_clus^seed then
                    add r_j to newClus;
                end
            end
        end
    end
end
if r_i ∈ C, CV_local(r_i) > 0.9/r_i ∈ C > threshold_buried then
    remove all residues r_i, CV_local(r_i) > 0.9 from R and
      restart clustering;
end
merge clusters < 5Å away from each other;
```

C, ensemble of detected clusters

$score(r_i)$, score of the residue r_i

$\mu(newClus + r_j)$, mean score computed over the ensemble of residues of cluster $newClus$ and r_j

$score^{seed}_{res}$, threshold score for residues during seeds detection

$score^{seed}_{clus}$, threshold score for clusters during seeds detection

$\{neighbors\ of\ newClus\}$, ensemble of residues < 5Å away from $newClus$

$r_i \in C, CV_{local}(r_i) > 0.9|/|r_i \in C$, percentage of residues in detected clusters with $CV_{local} > 0.9$

$threshold_{buried}$, maximum percentage of residues with $CV_{local} > 0.9$ admitted

Extension of a cluster c_k

```
for c_k ∈ C do
    scoreMax ← max_{r_i ∈ c_k}(score(r_i));
end
while μ(c_k) > score_clus^ext do
    newLayer_extension ← {};
    for r_j ∈ {neighbors of c_k} do
        if score_res^ext < score(r_j) < scoreMax then
            add r_j to newLayer_extension;
        end
    end
    add newLayer_extension to c_k;
    scoreMax ← max_{r_i ∈ newLayer_extension}(score(r_i));
end
while r_i ∈ C/r_i ∈ surface < 0.7 f_intfrac^DNA(x) do
    iteratively filter c_k ∈ C, where
      size(c_k) < size(c_{k+1})
end
if r_i ∈ C, CV_local(r_i) > 0.9/r_i ∈ C > threshold_buried
 then
    remove all residues r_i, CV_local(r_i) > 0.9 from R
      and restart clustering;
end
merge clusters < 5Åaway from each other;
```

$score(r_i)$, score of the residue r_i

$\mu(c_k)$, mean score computed over a cluster c_k

$score^{ext}_{res}$, threshold score for residues during extension step

$score^{ext}_{clus}$, threshold score for clusters during extension step

$\{neighbors\ of\ c_k\}$, ensemble of residues < 5Å away from c_k

$f^{DNA}_{intfrac}(x)$, expected size of the interface given x surface residues

Addition of an outer layer to a cluster c_k

```
outLayer ← {};
for c_k ∈ C do
    for r_j ∈ {neighbors of c_k} do
        if (μ(c_k) × |c_k| + score(r)) / (|c_k| + 1) ≥ μ(c_k) then
            add r_j to outLayer;
        end
    end
    add outLayer to c_k;
end
if r_i ∈ C/r_i ∈ surface < 0.7 f_intfrac^DNA(x) then
    relax score_res^seed, score_clus^seed, and score_clus^ext;
    restart clustering;
end
```

$\{neighbors\ of\ c_k\}$, ensemble of residues < 5Å away from c_k

Table D: **Confidence levels with which** $score_{res}$ **and** $score_{clus}$ **are determined in each layer.** The thresholds are in terms of $f^{DNA}_{intfrac}(x)$, that represents the expected size of a protein-DNA interface for a protein with x surface residues. Dashes mean that the threshold cannot be relaxed.

	Layer	First stage (not relaxed)	Second stage (relaxed)
$score_{res}$	*seed*	$f^{DNA}_{intfrac}(x)$	$2f^{DNA}_{intfrac}(x)$
	extension	$2f^{DNA}_{intfrac}(x)$	–
	outer layer	$2f^{DNA}_{intfrac}(x)$	–
$score_{clus}$	*seed*	$f^{DNA}_{intfrac}(x)/6$	$f^{DNA}_{intfrac}(x)/4$
	extension	$f^{DNA}_{intfrac}(x)/4$	$f^{DNA}_{intfrac}(x)/3$
	outer layer	$f^{DNA}_{intfrac}(x)/4$	$f^{DNA}_{intfrac}(x)/3$

Table E: **$\mathbf{JET^2_{DNA}}$ automated clustering procedure and completion by a second scoring scheme.**

First round to detect main clusters

choose **D-SC1**;
detect cluster seeds;
if $\mu(T_{JET}^{Seeds}) < 0.3$ **then**
  choose **D-SC3**;
  detect cluster seeds;
end
if *chosen* **D-SC1 then**
  if $\mu(CV_{glob}^{Seeds}) > 0.6$ *and* $\mu(PC_{DNA}^{Seeds}) < 0.9$ **then**
    choose **D-SC2**;
    detect cluster seeds;
  end
end
add the extensions to the seeds;
add the outer layers to the clusters;

• Test whether the evolutionary signal of detected seeds is too low:
$\mu(T_{JET}^{Seeds})$, mean value of evolutionary conservation over all seeds;

• Test whether seeds are located in a concave region of the protein surface and they do not have a very high average PC_{DNA} value:
$\mu(PC_{DNA}^{Seeds})$, mean value of propensities over all seeds;
$\mu(CV_{glob}^{Seeds})$, mean value of global circular variance computed with $r_c = 100$Å, over all seeds;

Second round to detect additional clusters using a complementary D-SC

if *chosen* **D-SC1** *or chosen* **D-SC2 then**
  choose **D-SC3**;
else
  choose **D-SC1**;
end
detect cluster seeds;
add the extension to the seeds;
add the outer layers to the clusters;
combine clusters;

Merge main clusters with additional clusters < 5Å away

Table F: **Comparison of iJET$^2_{\mathrm{DNA}}$, multiVORFFIP, DISPLAR and DRNApred performances on HR-PDNA187()(*)(**)(***) and APO82 datasets.** Statistical performance values are given in percentages. To fairly assess DISPLAR, multiVORFFIP and DRNApred performances, the proteins used for training these methods were removed from HR-PDNA187. We used a sequence identity cutoff of 95% and ended up with 106 (*), 87 (**) and 42 (***) proteins, respectively. iJET$^2_{\mathrm{DNA}}$ predictions were obtained from a consensus of 2 or 8 runs out of 10. The three scoring schemes were systematically used and the best patch or combination of patches was retained. For DISPLAR and DRNApred, binary prediction results were evaluated. For multiVORFFIP, predicted residues were defined as the ones with probability > 0.5. For each dataset, the best values are highlighted in bold. If multiple values in the same column of the same dataset are reported in bold, it means that their difference, if any, is not statistical significant (p-value ≥ 0.05).

	**Sens**	**PPV**	**Spe**	**Acc**	**F1**	**MCC**
**HR-PDNA187**						
iJET$^2_{\mathrm{DNA}}$(2/10)	**69**	58	86	84	**61**	**52**
iJET$^2_{\mathrm{DNA}}$(8/10)	63	**63**	**90**	**85**	**61**	**52**
**HR-PDNA187***						
iJET$^2_{\mathrm{DNA}}$(2/10)	**70**	57	85	84	**62**	**52**
iJET$^2_{\mathrm{DNA}}$(8/10)	64	**62**	89	**85**	**61**	**52**
DISPLAR	50	**62**	**92**	**85**	52	46
**HR-PDNA187****						
iJET$^2_{\mathrm{DNA}}$(2/10)	**71**	55	84	83	**60**	**50**
iJET$^2_{\mathrm{DNA}}$(8/10)	63	58	88	**84**	**59**	**49**
multiVORFFIP ($p > 0.5$)	44	**65**	**93**	**84**	50	43
**HR-PDNA187*****						
iJET$^2_{\mathrm{DNA}}$(2/10)	**70**	54	82	81	**60**	**48**
iJET$^2_{\mathrm{DNA}}$(8/10)	62	**59**	**87**	**82**	**59**	**48**
DRNApred	36	46	**86**	78	25	35
**APO82**						
iJET$^2_{\mathrm{DNA}}$(2/10)	**69**	54	88	86	**58**	**52**
iJET$^2_{\mathrm{DNA}}$(8/10)	63	58	92	**88**	**59**	**52**
DISPLAR	41	54	94	*87*	43	39
multiVORFFIP ($p > 0.5$)	44	**64**	**95**	**87**	50	45
DRNApred	23	40	**95**	85	24	21

Table G: **iJET$^2_{\mathbf{DNA}}$ performance on bound and unbound forms having the same or a different stoichiometry.** HOLO82$^+$ and APO82$^+$: 74 structures having the same stoichiometry in the HOLO and APO forms. HOLO82^{++} and APO82^{++}: 8 structures having a different stoichiometry in the HOLO and APO forms. Statistical performance values are given in percentages. The performance values are reported for the default (iJET$^2_{\mathrm{DNA}}$), the automated (iJET$^2_{\mathrm{DNA}\,Auto}$), the complete (iJET$^2_{\mathrm{DNA}\,Complete}$) and the automated+complete (iJET$^2_{\mathrm{DNA}\,AutoComplete}$) clustering procedure of the algorithm. Predictions were obtained from a consensus of 2, 5 or 8 runs out of 10. The three scoring schemes were systematically used and the best patch or best combination of patches was retained.

	**Consensus (/10 runs)**	**Sens**	**PPV**	**Spe**	**Acc**	**F1**	**MCC**
**HOLO82$^+$ (74)**							
iJET$^2_{\mathrm{DNA}}$	2	67	55	90	87	**59**	52
	5	64	58	91	87	**59**	53
	8	61	**60**	93	**88**	**59**	53
**APO82$^+$ (74)**							
iJET$^2_{\mathrm{DNA}}$	2	69	54	89	87	58	52
	5	66	55	90	87	**59**	52
	8	63	**57**	92	**88**	**59**	52
**HOLO82^{++} (8)**							
iJET$^2_{\mathrm{DNA}}$	2	70	55	82	79	**59**	47
	5	63	58	86	**81**	**59**	47
	8	60	**62**	89	**81**	**59**	48
**APO82^{++} (8)**							
iJET$^2_{\mathrm{DNA}}$	2	73	52	83	82	59	50
	5	68	57	88	84	**61**	52
	8	64	**59**	**90**	**84**	**60**	51

Table H: **Variation in iJET$^2_{DNA}$ performance when progressively adding layers in the clustering procedure, on bound and unbound forms.** Statistical performance values are reported for HR-PDNA187 and APO82 datasets and are given in percentages. iJET$^2_{DNA}$ predictions were obtained from a consensus of 2, 5 or 8 runs out of 10. The three scoring schemes were systematically used and the best patch or best combination of patches was retained. The performance values obtained when running predictions with an increasing number of layers in the clustering procedure are reported (1=*seed*; 2=*seed+extension*; 3 (default, also referred as iJET$^2_{DNA}$ in the other tables)=*seed+extension+outer layer*).

	**# layers**	**Consensus (/10 runs)**	**Sens**	**PPV**	**Spe**	**Acc**	**F1**	**MCC**
**HR-PDNA187**								
iJET$^2_{DNA}$	1	2	33	66	96	84	42	38
		5	28	69	97	84	38	36
		8	25	**71**	**98**	83	35	34
	2	2	46	63	93	84	51	43
		5	41	65	95	84	48	42
		8	37	67	96	84	45	41
	3	2	**69**	58	86	84	**61**	**52**
		5	66	61	88	**85**	**61**	**52**
		8	63	63	90	**85**	**61**	**52**
**APO82**								
iJET$^2_{DNA}$	1	2	30	60	97	87	38	36
		5	27	64	**98**	87	36	35
		8	24	**67**	**98**	87	33	34
	2	2	44	56	94	87	47	41
		5	40	61	96	87	46	41
		8	37	63	96	87	44	40
	3	2	**69**	54	**88**	86	58	**52**
		5	66	55	90	87	**59**	**52**
		8	63	58	92	**88**	**59**	**52**

Table I: **Comparison of iJET$^2_{DNA}$, multiVORFFIP, DISPLAR and DRNApred performances on TEST-NABP82, TEST-DBP49, TEST-RBP33 and TEST-DBP24 datasets.** Statistical performance values are given in percentages. To fairly assess DISPLAR, multiVORFFIP and DRNApred performances, TEST-DBP24 and TEST-RBP33 are non redundant at 25% to our HR-PDNA187 dataset. Moreover, all the TEST sets are evaluated on a different definiton of experimental interface, defined in [3], than the one used in HR-PDNA187. iJET$^2_{DNA}$ predictions were obtained from a consensus of 2 or 8 runs out of 10. The three scoring schemes were systematically used and the best patch or combination of patches was retained. DRNApred and multiVORFFIP were evaluated on the results provided by their RNA-specific algorithms for the RNA-binding proteins, and on the results provided by their DNA-specific algorithms for the DNA-binding proteins. iJET$^2_{DNA}$ and DISPLAR, which do not have a specific algorithm to predict RNA-binding sites, were evaluated on the same algorithm for both DNA- and RNA-binding proteins. For DISPLAR and DRNApred, binary prediction results were evaluated. For multiVORFFIP, predicted residues were defined as the ones with probability > 0.5. For each dataset, the best values are highlighted in bold. If multiple values in the same column of the same dataset are reported in bold, it means that their difference, if any, is not statistical significant (p-value ≥ 0.05).

	**Sens**	**PPV**	**Spe**	**Acc**	**F1**	**MCC**
**TEST-NABP82**						
iJET$^2_{DNA}$(2/10)	**67**	37	84	83	**45**	**39**
iJET$^2_{DNA}$(8/10)	59	**42**	89	**86**	**45**	**40**
DISPLAR	37	33	**91**	**86**	30	25
multiVORFFIP ($p > 0.5$)	46	37	88	**84**	37	31
DRNApred	23	20	**92**	**85**	16	13
**TEST-DBP49**						
iJET$^2_{DNA}$(2/10)	**66**	38	84	82	**45**	**39**
iJET$^2_{DNA}$(8/10)	59	**43**	88	**85**	**46**	**41**
DISPLAR	46	34	89	**85**	34	29
multiVORFFIP ($p > 0.5$)	49	**42**	89	**85**	41	**35**
DRNApred	30	28	**92**	**85**	23	19
**TEST-RBP33**						
iJET$^2_{DNA}$(2/10)	**68**	**36**	84	83	**44**	**39**
iJET$^2_{DNA}$(8/10)	58	**40**	89	**86**	**44**	**39**
DISPLAR	23	31	**94**	**87**	23	19
multiVORFFIP ($p > 0.5$)	42	29	86	82	31	24
DRNApred	12	8	**92**	**84**	6	3
**TEST-DBP24**						
iJET$^2_{DNA}$(2/10)	**64**	33	84	83	41	**36**
iJET$^2_{DNA}$(8/10)	57	**41**	90	**87**	**44**	**39**
DISPLAR	50	**36**	**89**	**85**	35	**31**
multiVORFFIP ($p > 0.5$)	**53**	**40**	89	**86**	**42**	**37**
DRNApred	25	22	**94**	**86**	16	14

Table J: **Variation in iJET$^2_{\mathrm{DNA}}$ performance when applying the default, the automated, the complete and the automated+complete clustering procedures, on bound and unbound forms.** Statistical performance values are reported for HR-PDNA187, HOLO82 and APO82 datasets and are given in percentages. The performance values are reported for the default (iJET$^2_{\mathrm{DNA}}$), the automated (iJET$^2_{\mathrm{DNA}\,Auto}$), the complete (iJET$^2_{\mathrm{DNA}\,Complete}$) and the automated+complete (iJET$^2_{\mathrm{DNA}\,AutoComplete}$) clustering procedure of the algorithm. Predictions were obtained from a consensus of 2, 5 or 8 runs out of 10. The three scoring schemes were systematically used and the best patch or best combination of patches was retained.

	**Consensus (/10 runs)**	**Sens**	**PPV**	**Spe**	**Acc**	**F1**	**MCC**
**HR-PDNA187**							
iJET$^2_{\mathrm{DNA}}$	2	69	58	86	84	**61**	**52**
	5	66	61	88	**85**	**61**	**52**
	8	63	**63**	90	**85**	**61**	**52**
iJET$^2_{\mathrm{DNA}\,Auto}$	2	59	53	87	82	54	44
	5	53	58	90	83	53	44
	8	46	62	**93**	84	50	43
iJET$^2_{\mathrm{DNA}\,Complete}$	2	**72**	50	81	80	57	46
	5	68	53	84	82	57	47
	8	64	54	86	82	57	47
iJET$^2_{\mathrm{DNA}\,AutoComplete}$	2	64	46	81	79	52	40
	5	58	49	85	80	51	40
	8	51	51	88	81	49	39
**HOLO82**							
iJET$^2_{\mathrm{DNA}}$	2	67	55	89	86	**59**	**52**
	5	65	58	91	**87**	**59**	**52**
	8	62	**61**	92	**87**	**59**	**52**
iJET$^2_{\mathrm{DNA}\,Auto}$	2	58	50	89	84	51	44
	5	52	55	92	86	51	44
	8	46	59	**94**	86	49	43
iJET$^2_{\mathrm{DNA}\,Complete}$	2	**70**	46	83	82	53	45
	5	67	48	85	83	54	48
	8	63	51	89	85	55	47
iJET$^2_{\mathrm{DNA}\,AutoComplete}$	2	62	43	84	81	49	39
	5	57	45	86	82	48	39
	8	52	47	89	83	48	39
**APO82**							
iJET$^2_{\mathrm{DNA}}$	2	69	54	88	86	58	**52**
	5	66	55	90	87	**59**	**52**
	8	63	**58**	92	**88**	**59**	**52**
iJET$^2_{\mathrm{DNA}\,Auto}$	2	61	49	89	85	52	44
	5	54	52	92	86	51	44
	8	47	55	**94**	87	49	43
iJET$^2_{\mathrm{DNA}\,Complete}$	2	**72**	44	83	82	53	45
	5	68	47	86	83	54	46
	8	64	49	88	85	54	47
iJET$^2_{\mathrm{DNA}\,AutoComplete}$	2	67	40	82	80	49	40
	5	60	43	86	82	48	39
	8	54	45	88	83	47	39

Table K: **F1 values of iJET$^2_{DNA}$, multiVORFFIP (MV), DISPLAR and DRNApred for proteins presenting multiple DNA-binding sites.** F1 values are given in percentage. iJET$^2_{DNA}$ predictions were obtained from a consensus of 2 or 5 runs out of 10, depending on the protein analyzed. The three scoring schemes were systematically used and performances for each of them as well as for the best patch or best combination of patches (B.C.P.) are given. For this latter case, the scoring schemes giving the best combination of patches are reported in square brackets. The performance values obtained when running the complete clustering procedure of the program are also given in round brackets, if the corresponding F1 value is higher than the default clustering procedure. For each case studied, the first row corresponds to the DNA-binding site occupied in the structure comprised in our HR-PDNA187 dataset, the second row corresponds to the DNA-binding site(s) occupied in the alternative structure, and the third row corresponds to the union of all DNA-binding residues in the two structures. For T7 RNA polymerase having a hybrid DNA+RNA binding site, we considered DNA and RNA-binding predictions for DRNApred and multiVORFFIP since they have DNA- and RNA-specific algorithms. White lines correspond to the performance related to the DNA-binding site(s) occupied in the structure reported on top of the same column. Grey lines correspond to the performance related to the mapped DNA-binding site(s) from the alternative structure in the column beside, and to the union of all DNA-binding residues from both the structures. For DISPLAR, multiVORFFIP (MV) and DRNApred, values in red mean that the tool was trained on that complex or on a homologous one showing a similar DNA-binding site. Bold values are the best ones excluding the red ones.

	**cplx in HR-PDNA187**							**alternative cplx**						
	**D-SC1**	**D-SC2**	**D-SC3**	**B.C.P.**	**MV**	**DISPLAR**	**DRNApred**	**D-SC1**	**D-SC2**	**D-SC3**	**B.C.P.**	**MV**	**DISPLAR**	**DRNApred**
	RNA polymerase from bacteriophage T7													
	**1CEZ (consensus 2/10 runs)**							**1MSW (consensus 2/10 runs)**						
**Site 1**	9	8	36	36 [D-SC3]	81	**39**	3	8	11	37	**41** [D-SC2+3]	35	25	3
**Site 2**	41 (46)	40	30 (0.52)	**55** [D-SC1+3]	33	37	8	43 (46)	42 (43)	19 (49)	**50** [D-SC1+3]	74	54	7
**Union all**	31 (36)	31	31 (47)	**49** [D-SC1+3]	60	44	5	34 (35)	35 (36)	26 (47)	**48** [D-SC1+2+3]	69	48	5
	Nuclease Domain of Adeno-Associated Virus Rep													
	**1UUT (consensus 5/10 runs)**							**1RZ9 (consensus 5/10 runs)**						
**Site 1**	44	9 (10)	30 (38)	**45** [D-SC1+3]	87	0	0	12 (14)	9	33	33 [D-SC3]	**44**	6	0
**Site 2**	**86**	4 (15)	62 (84)	**86** [D-SC1]	0	73	36	54	8 (35)	**73**	**73** [D-SC3]	63	67	36
**Union all**	**73**	10 (20)	49 (68)	**73** [D-SC1]	49	56	23	52	13 (37)	58	58 [D-SC3]	**78**	54	23
	Type 2 Restriction Endonuclease DpnI													
	**4ESJ (consensus 2/10 runs)**							**4KYW (consensus 2/10 runs)**						
**Site 1**	38	16 (24)	31 (33)	**40** [D-SC1+3]	32	34	19	**52**	3 (15)	43 (**52**)	**52** [D-SC1]	31	5	19
**Site 1+2**	64	50 (57)	34 (61)	**67** [D-SC1+3]	51	33	28	57 (65)	44 (62)	47 (**69**)	66 [D-SC1+2+3]	53	16	27
**Union all**	63	50 (58)	36 (62)	**68** [D-SC1+3]	55	38	29	55 (63)	45 (62)	45 (**67**)	65 [D-SC1+2+3]	54	17	28
	Cyclic GMP-AMP synthase													
	**4K98 (consensus 5/10 runs)**							**4LEY (consensus 5/10 runs)**						
**Site 1**	0 (6)	15 (17)	20	22 [D-SC2+3]	10	**35**	16	6 (7)	24	18	29 [D-SC2+3]	28	**30**	15
**Site 1+2**	27	10	**36**	**36** [D-SC3]	35	28	22	18 (20)	40	29	44 [D-SC2+3]	**50**	24	22
**Union all**	25	12 (14)	**37**	**37** [D-SC3]	32	26	21	17 (20)	42	27	45 [D-SC2+3]	**47**	26	20

Table L: Comparison of HR-PDNA187 and HOLO-APO82 with the most used datasets in previous studies.

Datasets	#(cplx)	Resol.(Å)	Seq. Id.(%)	#(APO forms)	#(shared cplx with HR-PDNA187)	#(shared APO forms with APO82)	#(flawed cplx)*	#(cplx after PISCES filtering)***
HR-PDNA187	187	2.5	25	86	-	-	-	
PDNA62 [4, 5]	62	3.0	25	0	0	5/62	2/62	27
DBP374 [6]	374**	3.5	25	0	0	36/374	6/374	121
MetaDBsite316 [7]	316**	3.0	30	0	0	53/316	7/316	139
Displar264 (chains) [8]	264	NA	50	0	0	52/264	12/264	124
PDDB1.2 [9]	47	3.0	60	47	8/47	8/47	0/47	28
DBP206 [10]	206**	3.0	25	83	85/206	6/83	9/206	141
PDNA224 [11]	224**	3.0	25	0	0	46/224	7/224	128
DNABINDPROT54 [12]	54**	NA	NA	54	9/54	10/54	1/54	30

* : complexes present in the analyzed dataset, but removed from HR-PDNA187 because the asymmetric unit does not contain at least one biological unit.
**: number of non redundant chains, instead of complexes.
***: number of complexes after PISCES filtering fixing the same parameters used to filter HR-PDNA187
