# Supplementary material for: Multiple protein-DNA interfaces unravelled by evolutionary information, physico-chemical and geometrical properties
Source: PLoS Comput Biol. 2020 Feb 3;16(2):e1007624. doi: 10.1371/journal.pcbi.1007624 (PMC7018136; doi:10.1371/journal.pcbi.1007624)
Supplement: S2 File — (PDF) [file pcbi.1007624.s002.pdf]

Supplementary Figures

References

[1] B. Park, H. Kim, and K. Han, "Dbbp: database of binding pairs in protein-nucleic acid interactions," *BMC Bioinformatics*, vol. 15, no. 15, p. S5, 2014.

[2] S. S. Negi and W. Braun, "Statistical analysis of physical-chemical properties and prediction of protein-protein interfaces," *Journal of Molecular Modeling*, vol. 13, no. 11, pp. 1157–1167, 2007.

[3] J. Kyte and R. F. Doolittle, "A simple method for displaying the hydropathic character of a protein," *Journal of Molecular Biology*, vol. 157, no. 1, pp. 105 – 132, 1982.

[4] S. Engelen, L. A. Trojan, S. Sacquin-Mora, R. Lavery, and A. Carbone, "Joint evolutionary trees: a large-scale method to predict protein interfaces based on sequence sampling," *PLoS Computational Biology*, vol. 5, no. 1, p. e1000267, 2009.

[5] H. Chen and H.-X. Zhou, "Prediction of interface residues in protein–protein complexes by a consensus neural network method: test against nmr data," *Proteins: Structure, Function, and Bioinformatics*, vol. 61, no. 1, pp. 21–35, 2005.

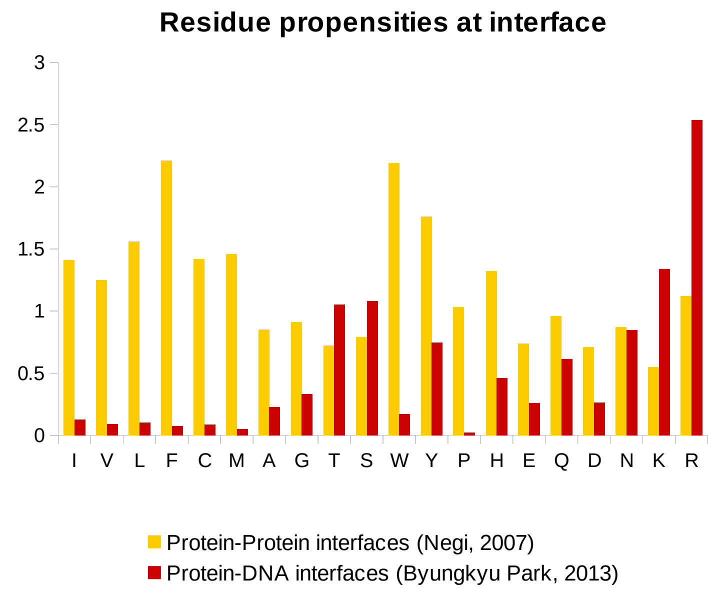

Figure A: **Interface propensities.** The protein-DNA (in red, [1]) and protein-protein (in orange, [2]) scales are shown on the same plot for visual comparison. Amino acids are ordered from the most to the least hydrophobic one, based on the Kyte and Doolittle hydrophobicity scale [3].

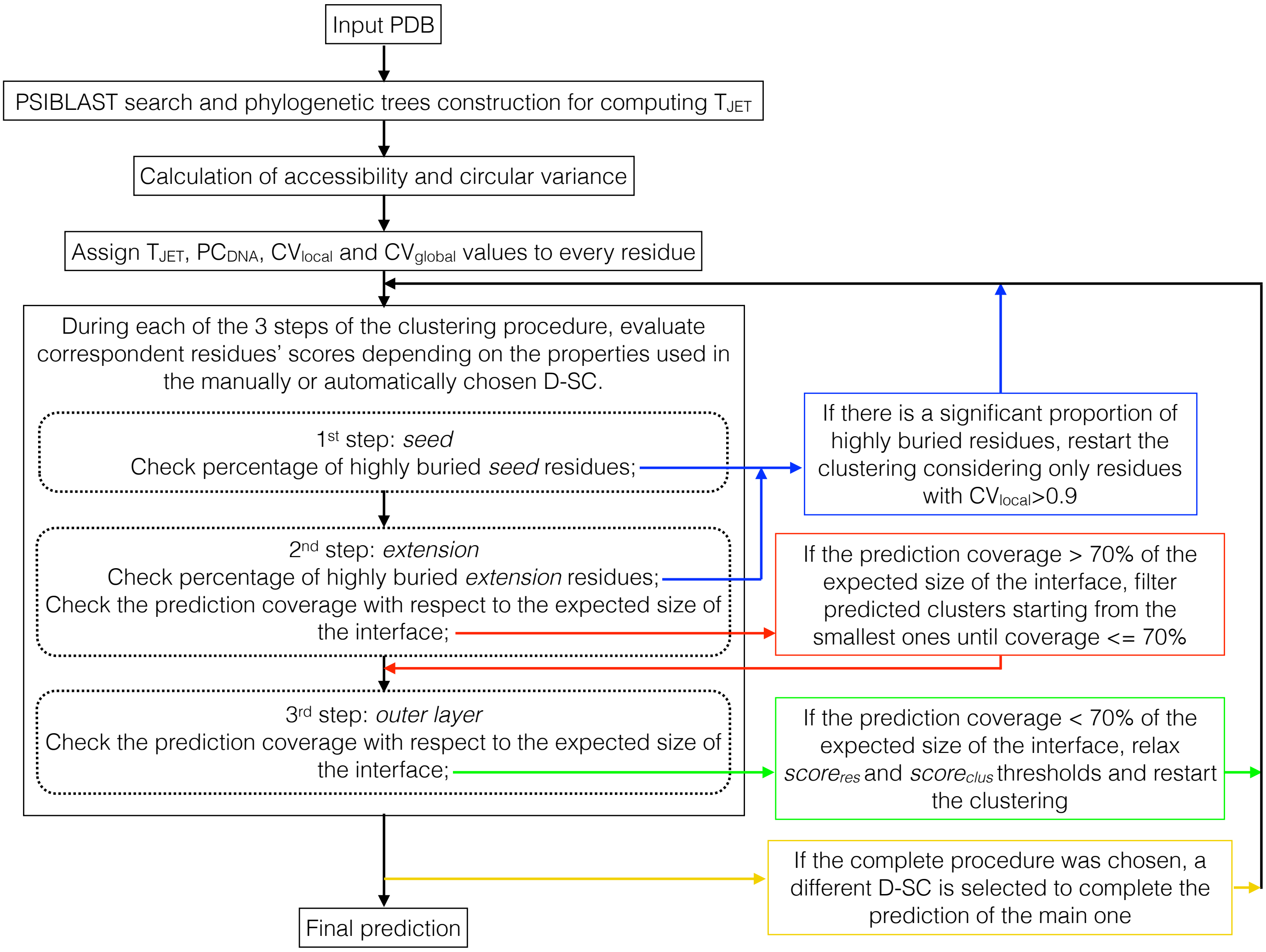

Figure B: **$\mathbf{JET^2_{DNA}}$ pipeline.** General JET^2_{DNA} pipeline, whatever the scoring scheme chosen. In black, the mandatory steps. In blue, procedure to avoid buried small ligand binding pockets. In red, filtering of the putative false positive clusters. In green, relaxing of the thresholds for too small predicted clusters, which do not respect the expecting size of the interface. In yellow, the possibility to complete the main prediction with a second one obtained by a different scoring scheme.

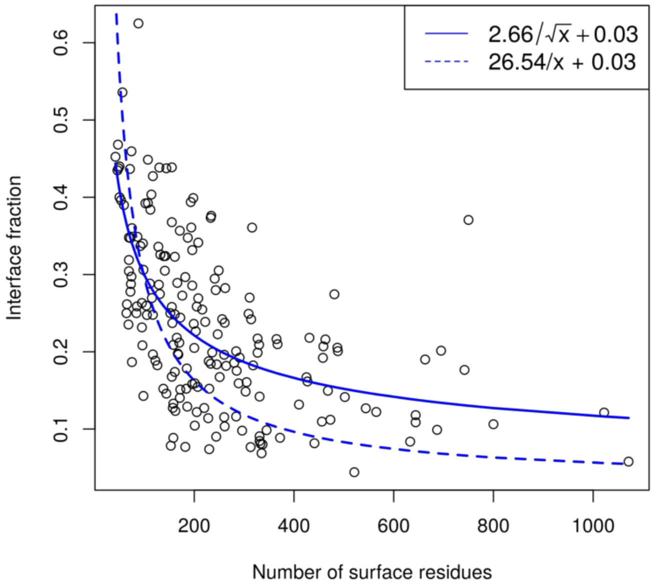

Figure C: **Expected size of the interface**. Plot of the $f_{intfrac}$ function relating surface size and fraction of the surface covered by the interface. Each circle is the percentage of interface residues versus the total number of surface residues for a given protein comprised in the HR-PDNA187 dataset. The solid line corresponds to the function that best approximates the circles distribution. The dotted line corresponds to the function that best approximates percentage of interface residues versus the total number of surface residues for protein-protein interfaces [4] based on a dataset of 1256 protein chains [5].

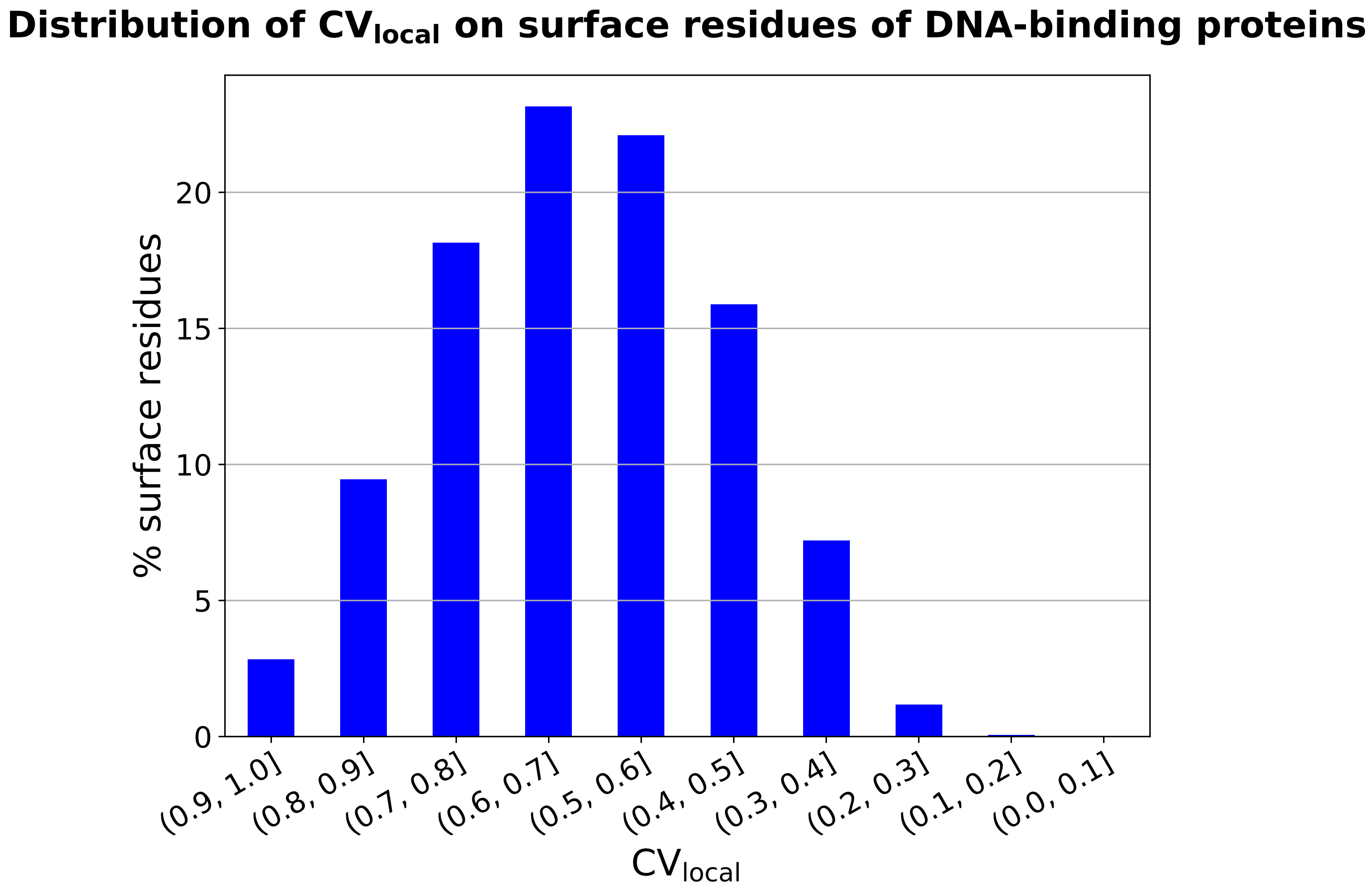

Figure D: **Distribution of CV_{local} values on DNA-binding protein surfaces.** Histogram of the percentage of surface residues having a CV_{local} value comprised in one of the ten intervals between [0, 1]. The calculation was performed on the HR-PDNA187 dataset. Surface residues are defined as the ones showing a relative solvent accessibility $\geq 5\%$. High values of CV_{local} indicate locally buried residues.

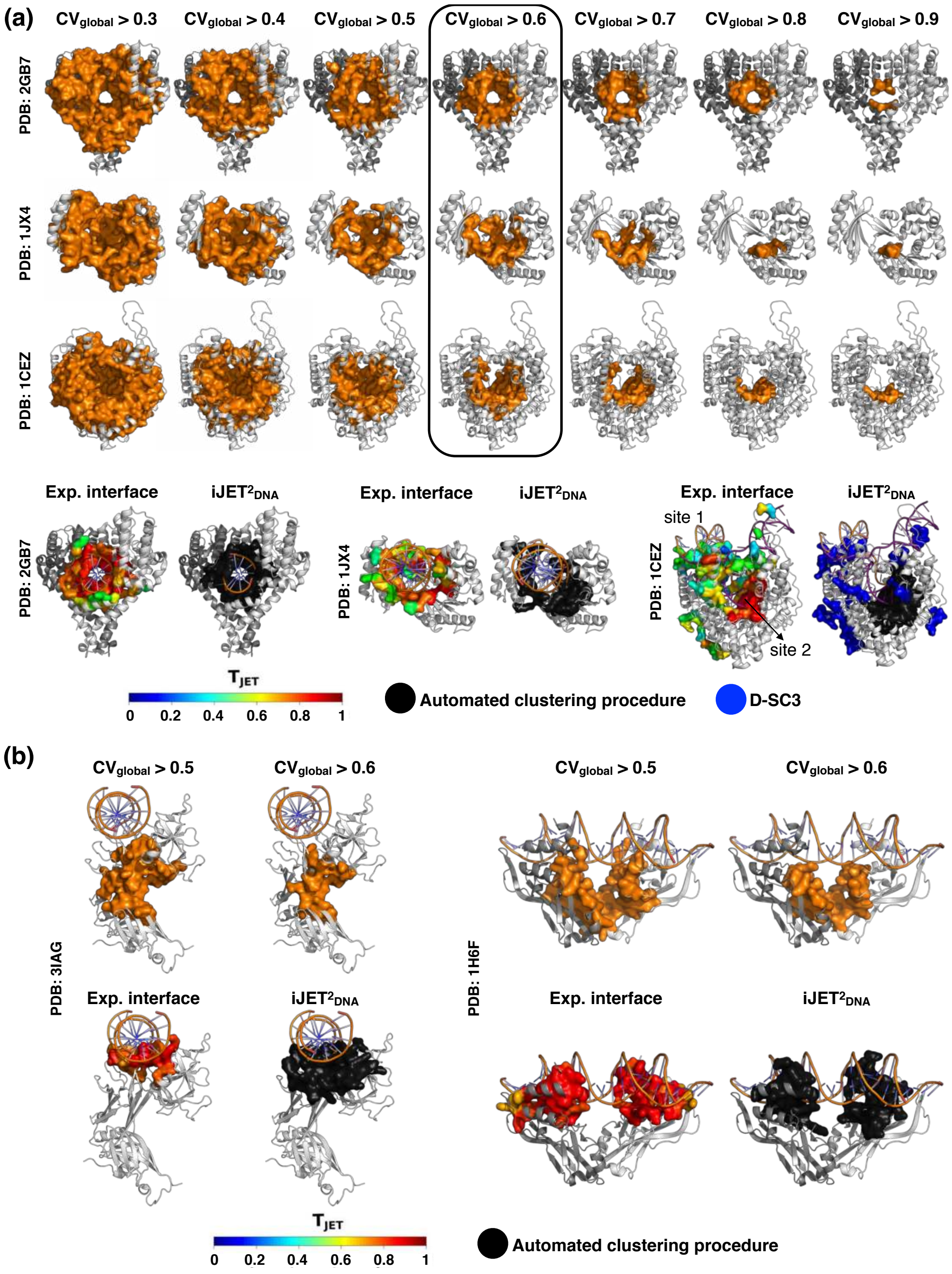

Figure E: **Examples of concave regions detected by $\mathrm{CV_{global}}$ and predictions by $\mathrm{JET^2_{DNA}}$ automated clustering procedure.** (a) Top: three examples of concave protein-DNA interfaces that are well detected using the $\mathrm{CV_{global}}$ descriptor. In each column, the ensemble of residues having a $\mathrm{CV_{global}}$ above the fixed threshold are represented as orange opaque surfaces. The three cases corresponding to the default threshold used in $\mathrm{JET^2_{DNA}}$ are highlighted in the black box. Bottom: experimentally validated DNA-binding site(s) and $\mathrm{JET^2_{DNA}}$ predictions of the three examples in (a) top. Experimental DNA-binding site(s) are colored according to $\mathrm{T_{JET}}$values. $\mathrm{JET^2_{DNA}}$ predictions were obtained running the automated clustering procedure (black opaque surfaces) and DSC3 (blue opaque surfaces), with a consensus of 2 runs out of 10. For PDB 1CEZ, the second DNA-binding site (site 2) was obtained mapping the DNA of PDB 1MSW (in purple) on the 1CEZ structure. (b) Top: two examples of concave protein regions showing no overlap with the DNA-binding sites and the detection of concavity by the $\mathrm{CV_{global}}$ descriptor. The ensemble of residues having a $\mathrm{CV_{global}}$ above 0.5 and 0.6 are represented as orange opaque surfaces. Bottom: experimentally validated DNA-binding site(s) and $\mathrm{JET^2_{DNA}}$ predictions of the two examples in (b) top. Experimental DNA-binding site(s) are colored according to $\mathrm{T_{JET}}$values. $\mathrm{JET^2_{DNA}}$ predictions were obtained running the automated clustering procedure (black opaque surfaces), with a consensus of 2 runs out of 10.

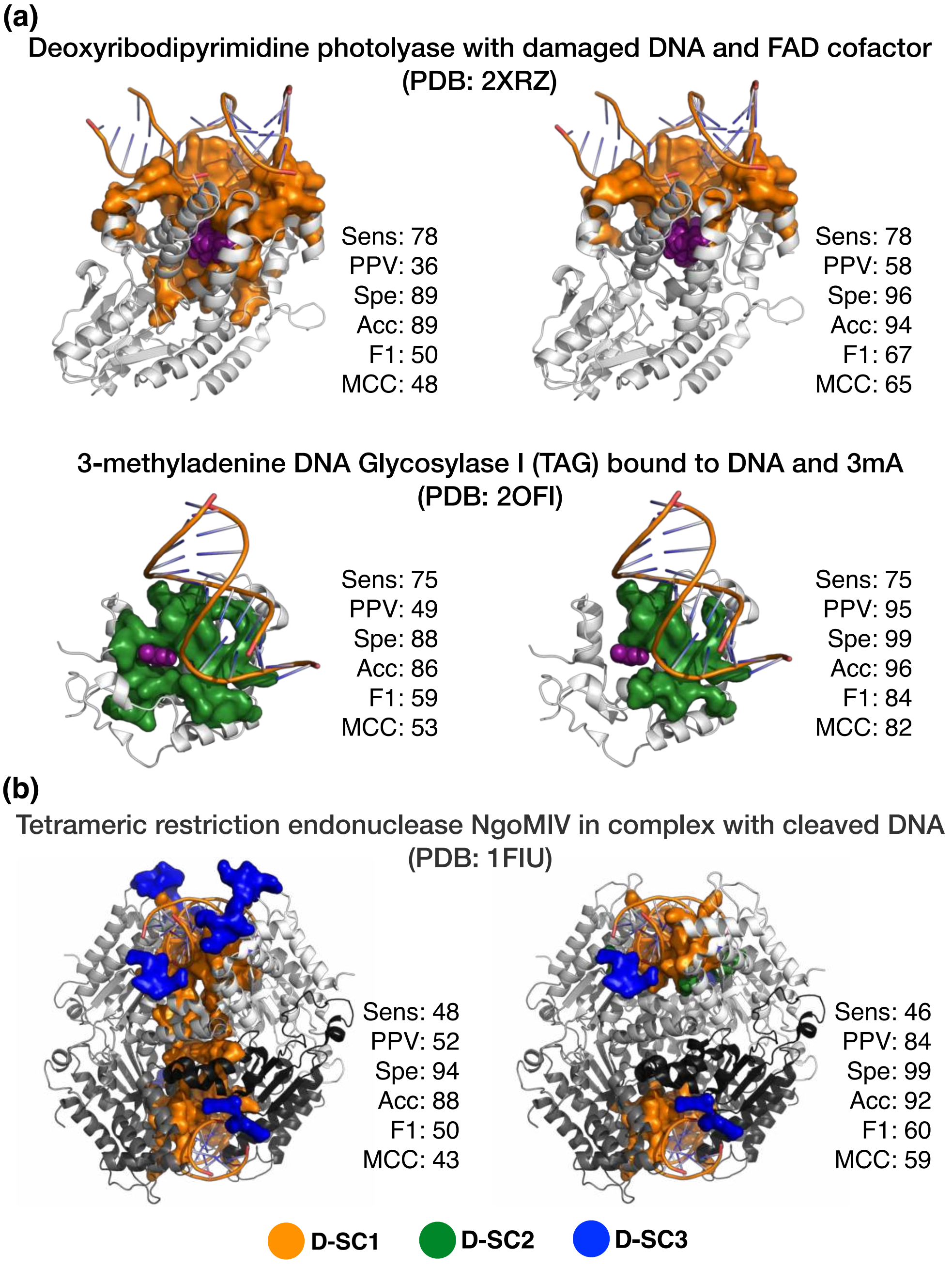

Figure F: **Examples of improvements in $\mathrm{JET}^2_{\mathrm{DNA}}$ predictions by the implementation of the procedure to avoid small ligand binding pockets.** On the left, $\mathrm{JET}^2_{\mathrm{DNA}}$ predictions without the procedure to avoid small ligand binding pockets. On the right, $\mathrm{JET}^2_{\mathrm{DNA}}$ predictions with the procedure using the default values reported in the paper ($\mathrm{CV}_{\mathrm{local}} = 0.9$ and $\%\ residues_{allowed} = 20\%$ (D-SC1), 30% (D-SC2)). (a) Two examples showing an improvement in the $\mathrm{JET}^2_{\mathrm{DNA}}$ prediction for both D-SC1 and D-SC2 by avoiding the prediction of the small ligand binding pocket close to the DNA-binding site. On top, a deoxyribodipyrimidine photolyase in complex with a damaged duplex DNA and a FAD cofactor (represented with purple spheres) (PDB code: 2XRZ). On bottom, the 3-methyladenine DNA Glycosylase I (TAG) in complex with abasic duplex DNA and a 3mA nucleobase (represented with purple spheres) (PDB code: 2OFI). (b) An example showing the procedure implemented to avoid small ligand binding pockets can ameliorate predictions on multimeric protein structures. The tetrameric restriction endonuclease NgoMIV in complex with two duplex DNA (PDB code: 1FIU) is reported with different protein chains greyscale colored. $\mathrm{JET}^2_{\mathrm{DNA}}$ predictions by D-SC1, D-SC2 and D-SC3 are represented as orange, green and blue opaque colored surfaces, respectively. All the predictions were obtained from a consensus of 2 runs out of 10 of $\mathrm{JET}^2_{\mathrm{DNA}}$.

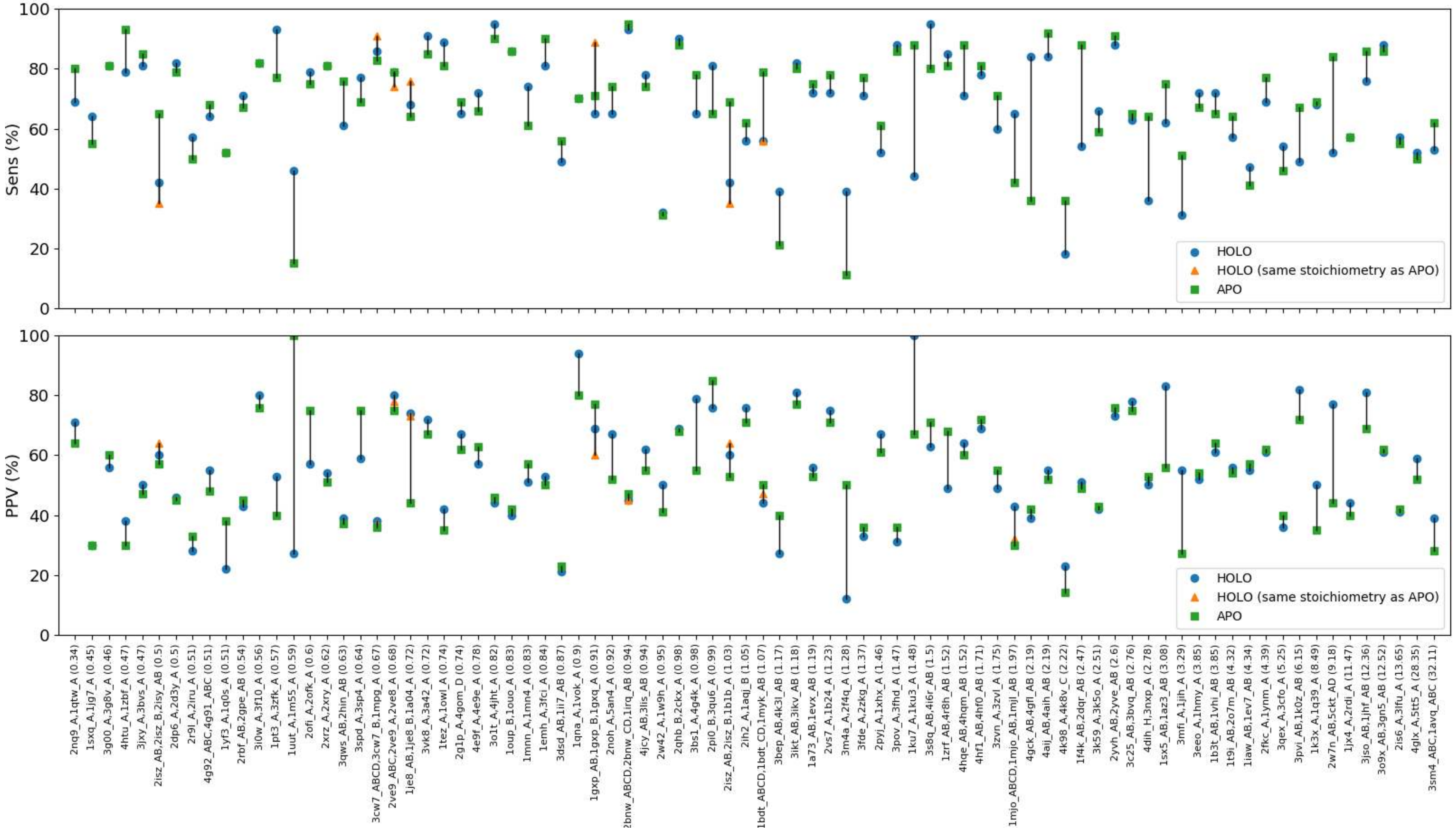

Figure G: **Variation in sensitivity and PPV values between predictions on HOLO and APO forms.** On the x-axis are reported the 82 HOLO-APO pairs (HOLO-APO82 dataset), ordered by the increasing RMSD between bound and unbound conformations of each pair. For the 8 pairs showing a HOLO form in a different stoichiometry (more protein chains) than the APO one, the subset of chains in the HOLO form corresponding to the same stoichiometry of the APO one is also reported. The format for the 74 HOLO-APO pairs in the same stoichiometry is "HOLO, APO (RMSD HOLO-APO)", while the format for the 8 HOLO-APO pairs in a different stoichiometry is "HOLO, HOLO_subsetChains, APO (RMSD HOLO-APO)". On the y-axis, sensitivity and PPV values are reported in percentage. Performance on HOLO, HOLO_subsetChains and APO forms are reported in blue circles, orange triangles and green squares, respectively. iJET$^2_{\mathrm{DNA}}$ predictions were obtained with a consensus of 2 runs out of 10.

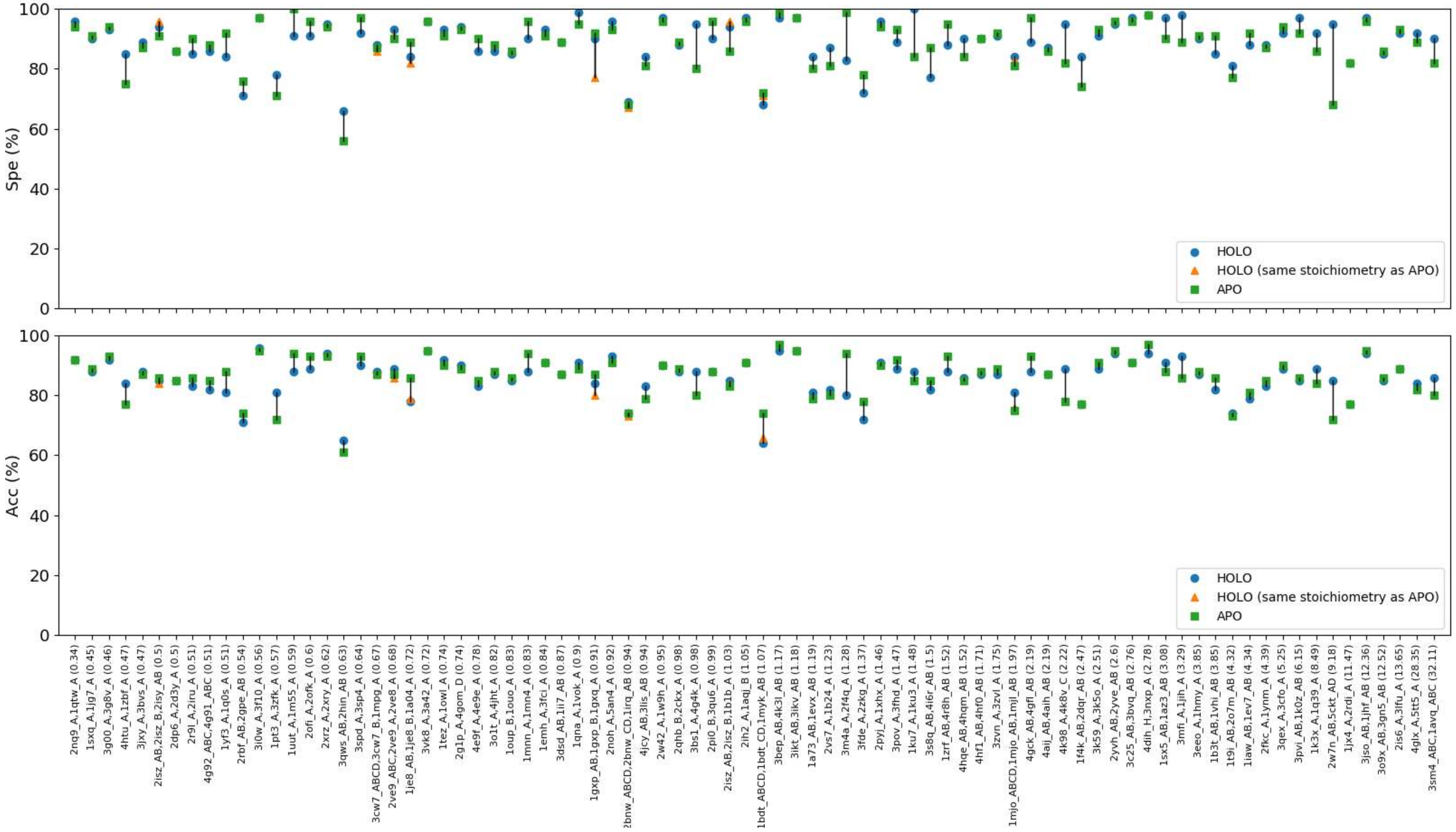

Figure H: **Variation in specificity and accuracy between predictions on HOLO and APO forms.** On the x-axis are reported the 82 HOLO-APO pairs (HOLO-APO82 dataset), ordered by the increasing RMSD between bound and unbound conformations of each pair. For the 8 pairs showing a HOLO form in a different stoichiometry (more protein chains) than the APO one, the subset of chains in the HOLO form corresponding to the same stoichiometry of the APO one is also reported. The format for the 74 HOLO-APO pairs in the same stoichiometry is "HOLO, APO (RMSD HOLO-APO)", while the format for the 8 HOLO-APO pairs in a different stoichiometry is "HOLO, HOLO_subsetChains, APO (RMSD HOLO-APO)". On the y-axis, specificity and accuracy values are reported in percentage. Performance on HOLO, HOLO_subsetChains and APO forms are reported in blue circles, orange triangles and green squares, respectively. iJET$^2_{\mathrm{DNA}}$ predictions were obtained with a consensus of 2 runs out of 10.

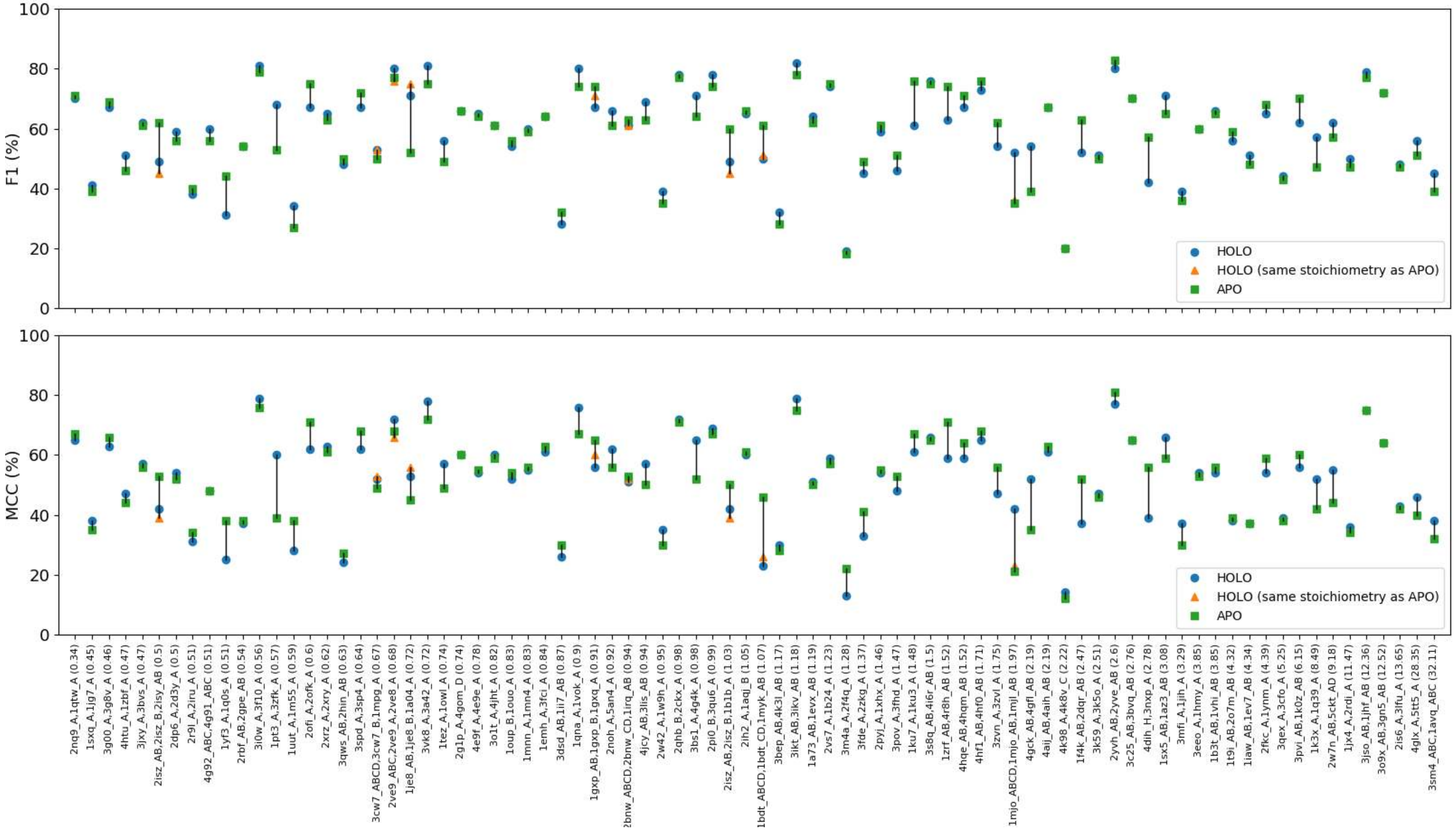

Figure I: **Variation in F1 and MCC values between predictions on HOLO and APO forms.** On the x-axis are reported the 82 HOLO-APO pairs (HOLO-APO82 dataset), ordered by the increasing RMSD between bound and unbound conformations of each pair. For the 8 pairs showing a HOLO form in a different stoichiometry (more protein chains) than the APO one, the subset of chains in the HOLO form corresponding to the same stoichiometry of the APO one is also reported. The format for the 74 HOLO-APO pairs in the same stoichiometry is "HOLO, APO (RMSD HOLO-APO)", while the format for the 8 HOLO-APO pairs in a different stoichiometry is "HOLO, HOLO_subsetChains, APO (RMSD HOLO-APO)". On the y-axis, F1 and MCC values are reported in percentage. Performance on HOLO, HOLO_subsetChains and APO forms are reported in blue circles, orange triangles and green squares, respectively. iJET$^2_{\mathrm{DNA}}$ predictions were obtained with a consensus of 2 runs out of 10.

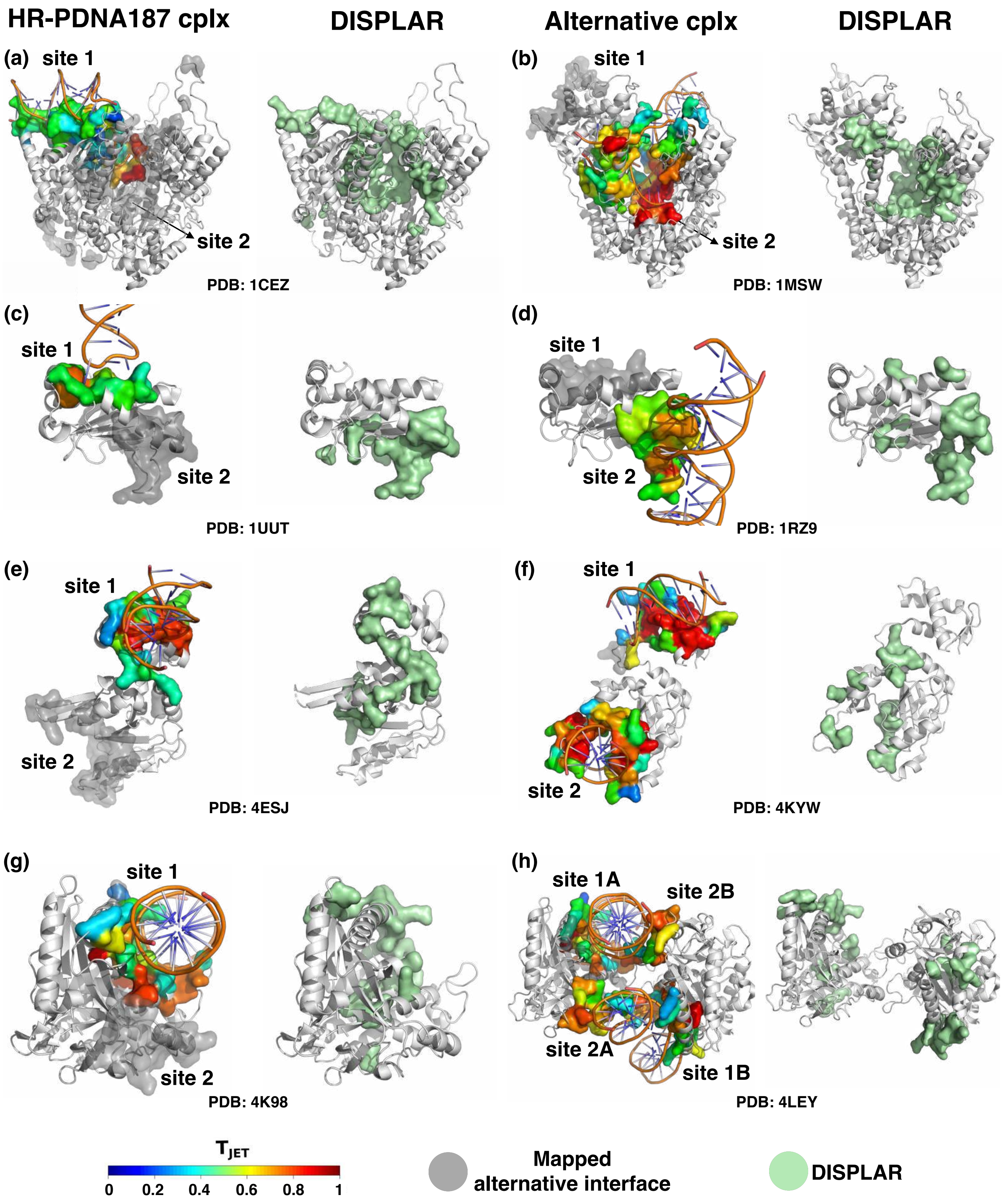

Figure J: **DISPLAR predictions on proteins presenting multiple DNA-binding sites.** DISPLAR predicted patches were defined as formed by residue indicated as predicted in the tool results and are colored in lightgreen. Compare with Fig. 5.

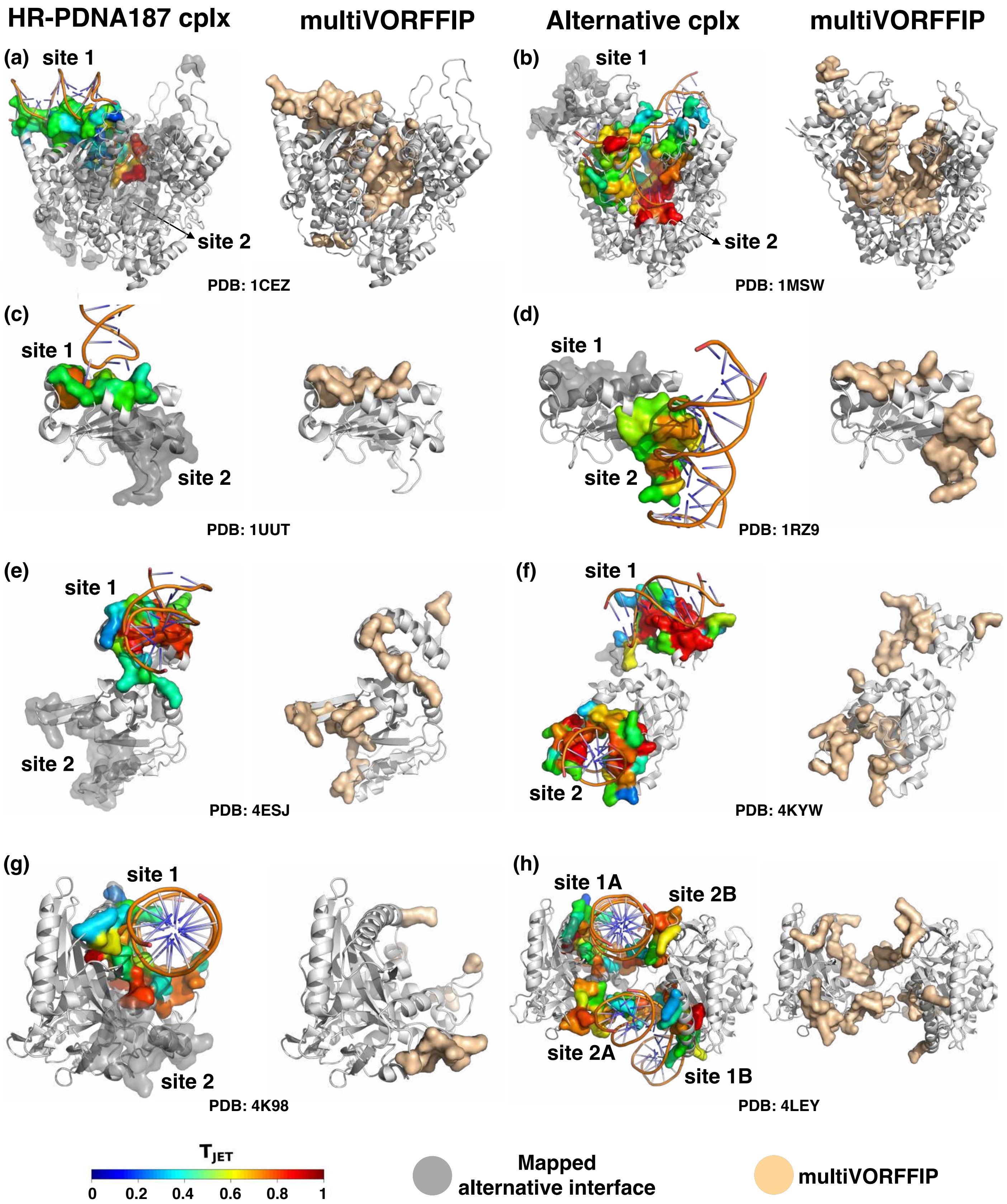

Figure K: **multiVORFFIP predictions on proteins presenting multiple DNA-binding sites.** multiVORFFIP predicted patches were defined as formed by residues with probability > 0.5 and are colored in beige. Compare with Fig. 5.

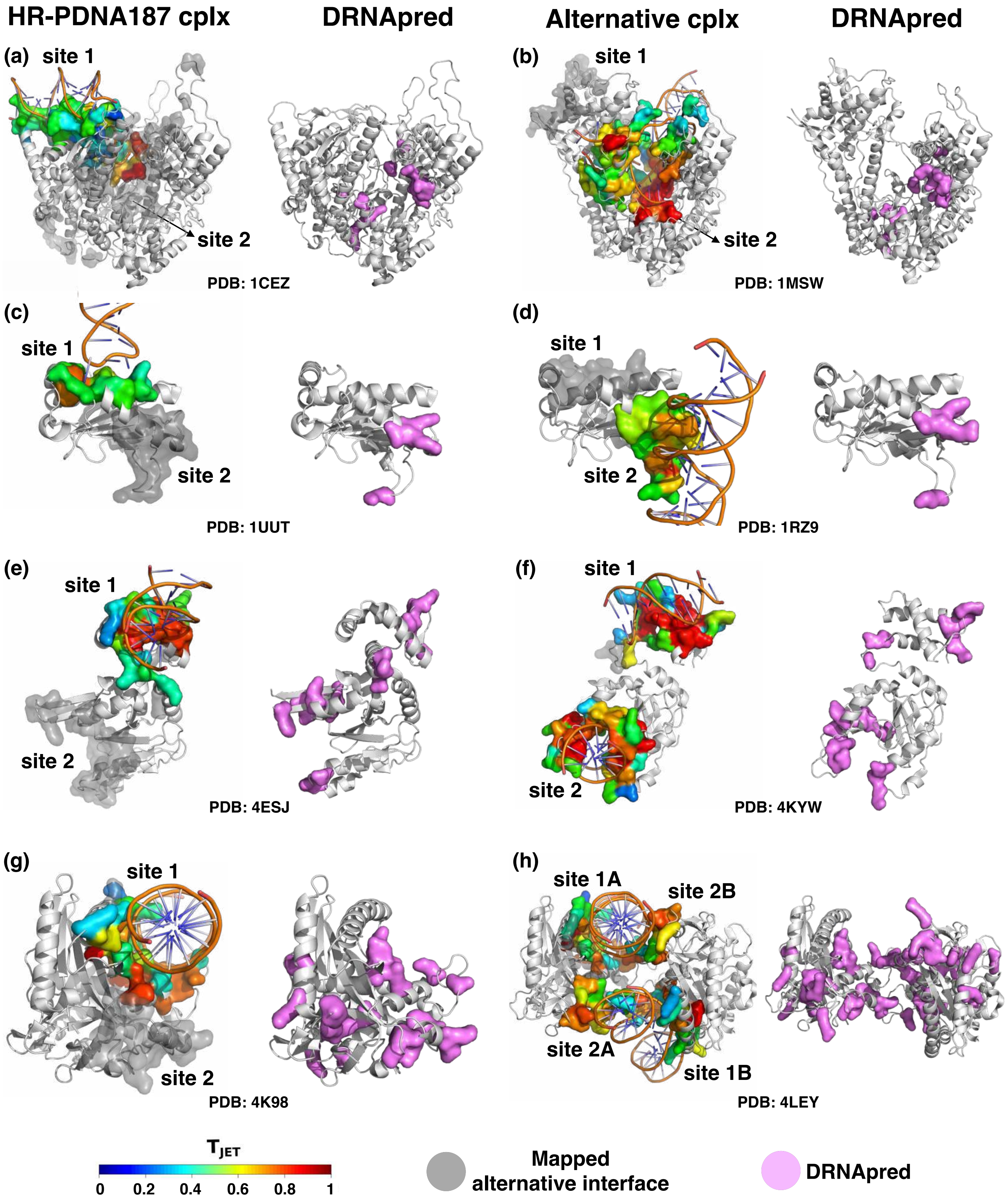

Figure L: **DRNApred predictions on proteins presenting multiple DNA-binding sites.** DRNApred predicted patches were defined as formed by residues indicated as predicted by the binary prediction column in the tool results and are colored in orchid. Compare with Fig. 5.

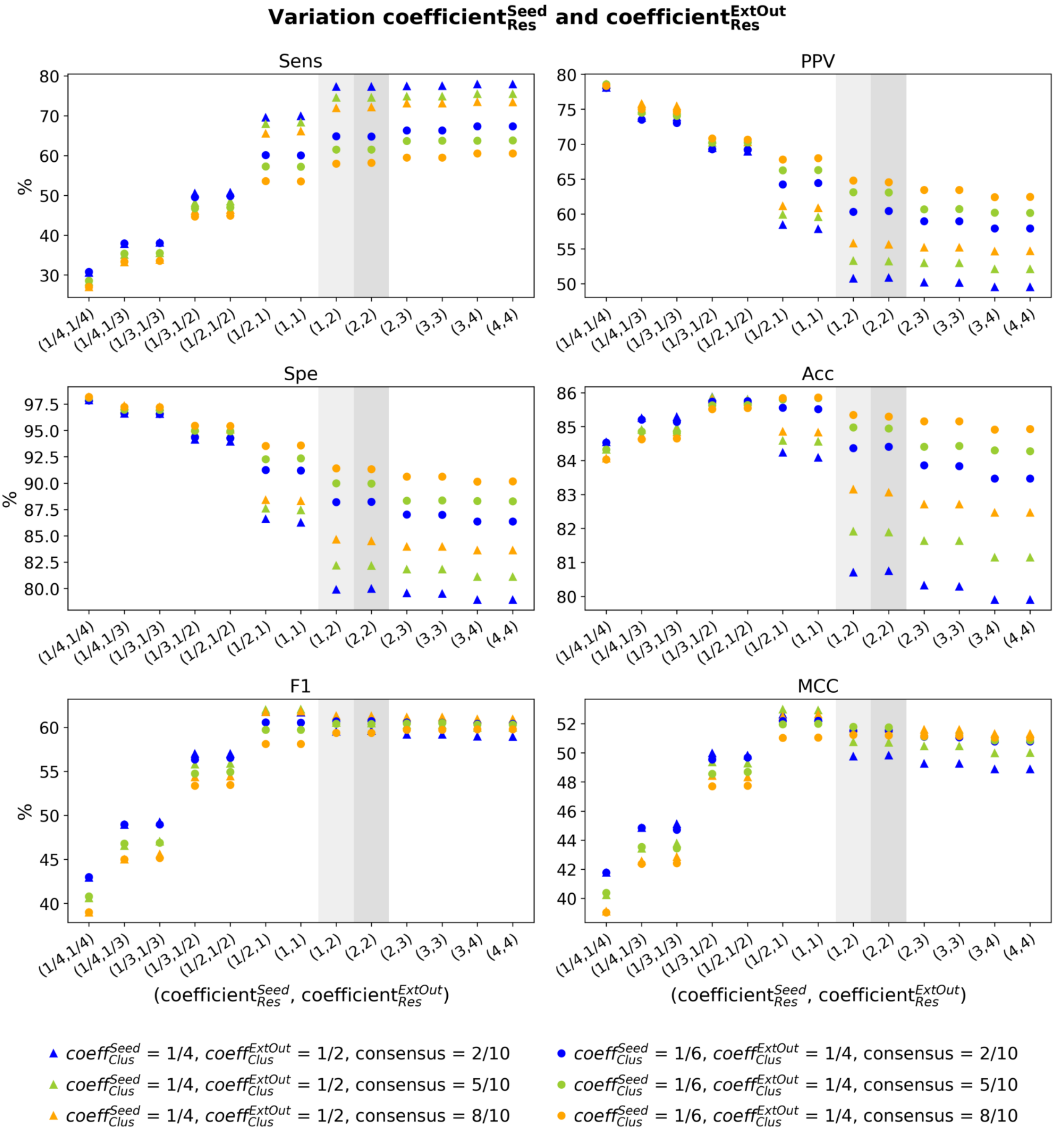

Figure M: **Influence of the two parameters coefficient$^{Seed}_{Res}$ and coefficient$^{ExtOut}_{Res}$ on the predictive performance of JET$^2_{\mathrm{DNA}}$.** These coefficients are multiplied by $f_{intfrac}$ to determine the two residue thresholds score$^{Seed}_{Res}$ and score$^{ExtOut}_{Res}$. Namely, only residues showing a score $\geq$ score$^{Seed}_{Res}$ (resp. score$^{ExtOut}_{Res}$) are considered by the JET$^2_{\mathrm{DNA}}$ algorithm during the detection of the seed (resp. extension). Two different sets of coefficient$^{Seed}_{Clus}$, coefficient$^{ExtOut}_{Clus}$ and Confidence$_{ClusterSize}$ were tried (triangles and circles) to test the stability of the results with respect to the other thresholds used in the algorithm. JET$^2_{\mathrm{DNA}}$ predictions obtained with a consensus of 2, 5 and 8 runs out of 10 are reported in blue, green and orange respectively. The calculation was performed on the HR-PDNA187 dataset. Default values chosen in JET$^2_{\mathrm{DNA}}$ for the first stage (not-relaxed values) and the second stage (relaxed values) of prediction are highlighted in lightgrey and grey, respectively.

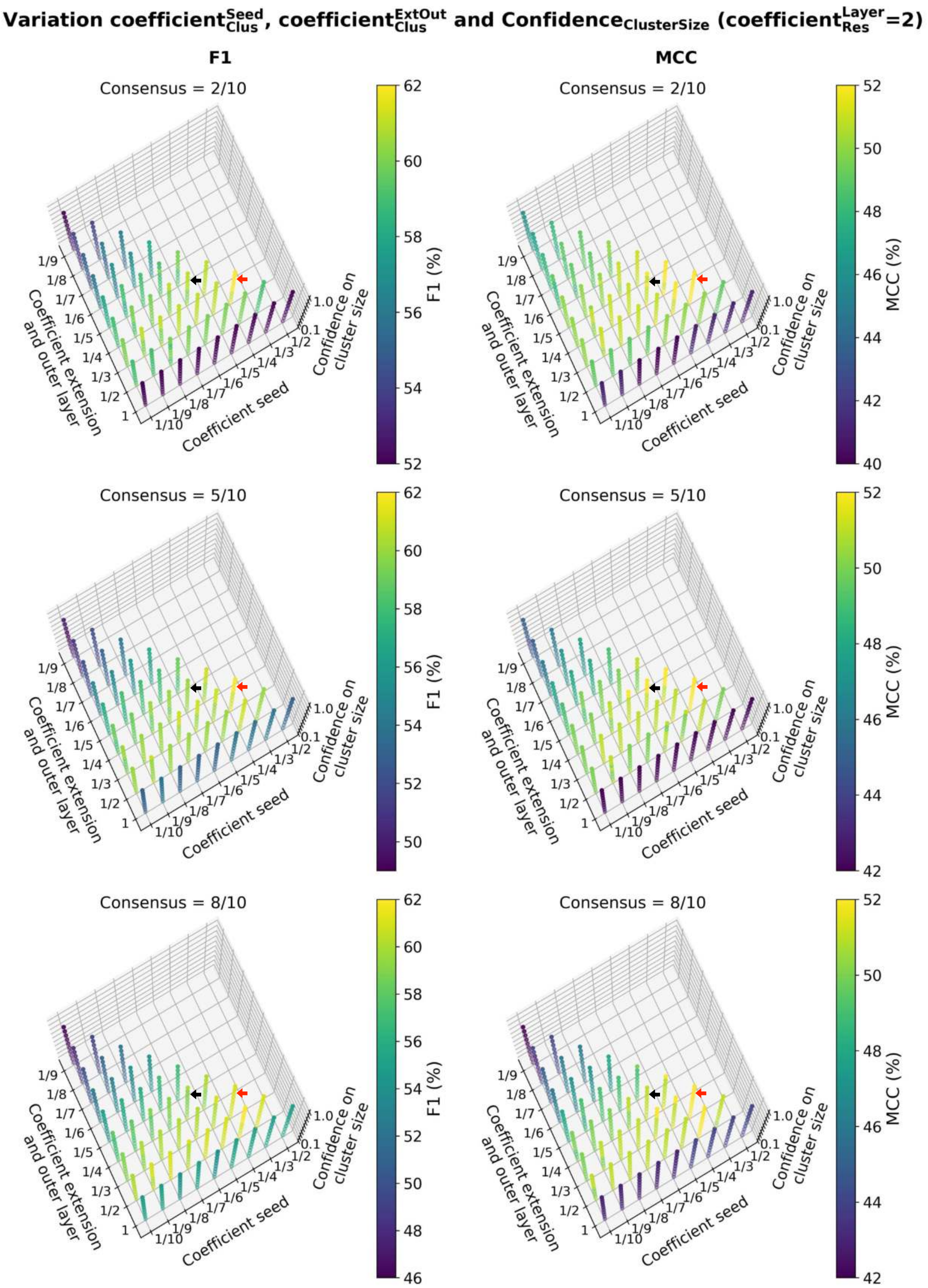

Figure N: **Influence of the three parameters coefficient$^{Seed}_{Clus}$, coefficient$^{ExtOut}_{Clus}$ and confidence$_{clusSize}$ on MCC and F1 values.** coefficient$^{Seed}_{Clus}$ and coefficient$^{ExtOut}_{Clus}$ are multiplied by $f_{intfrac}$ to determine the two cluster thresholds score$^{Seed}_{Clus}$ and score$^{ExtOut}_{Clus}$. Namely, a given seed (resp. extension) is grown until the averaged score of the predicted cluster reaches down score$^{Seed}_{Clus}$ (resp. score$^{ExtOut}_{Clus}$). The third threshold represents the confidence on the cluster size (confidence$_{clusSize}$). Clusters with a size below this confidence level are filtered out. The statistical value is coded by the color. Results obtained from a consensus of 2, 5 and 8 runs out of 10 are reported on top, at the center and bottom, respectively. The calculation was performed on the HR-PDNA187 dataset. Default values chosen in JET$^2_{\mathrm{DNA}}$ for the first stage (not-relaxed values) and the second stage (relaxed values) of prediction are highlighted in black and red, respectively.

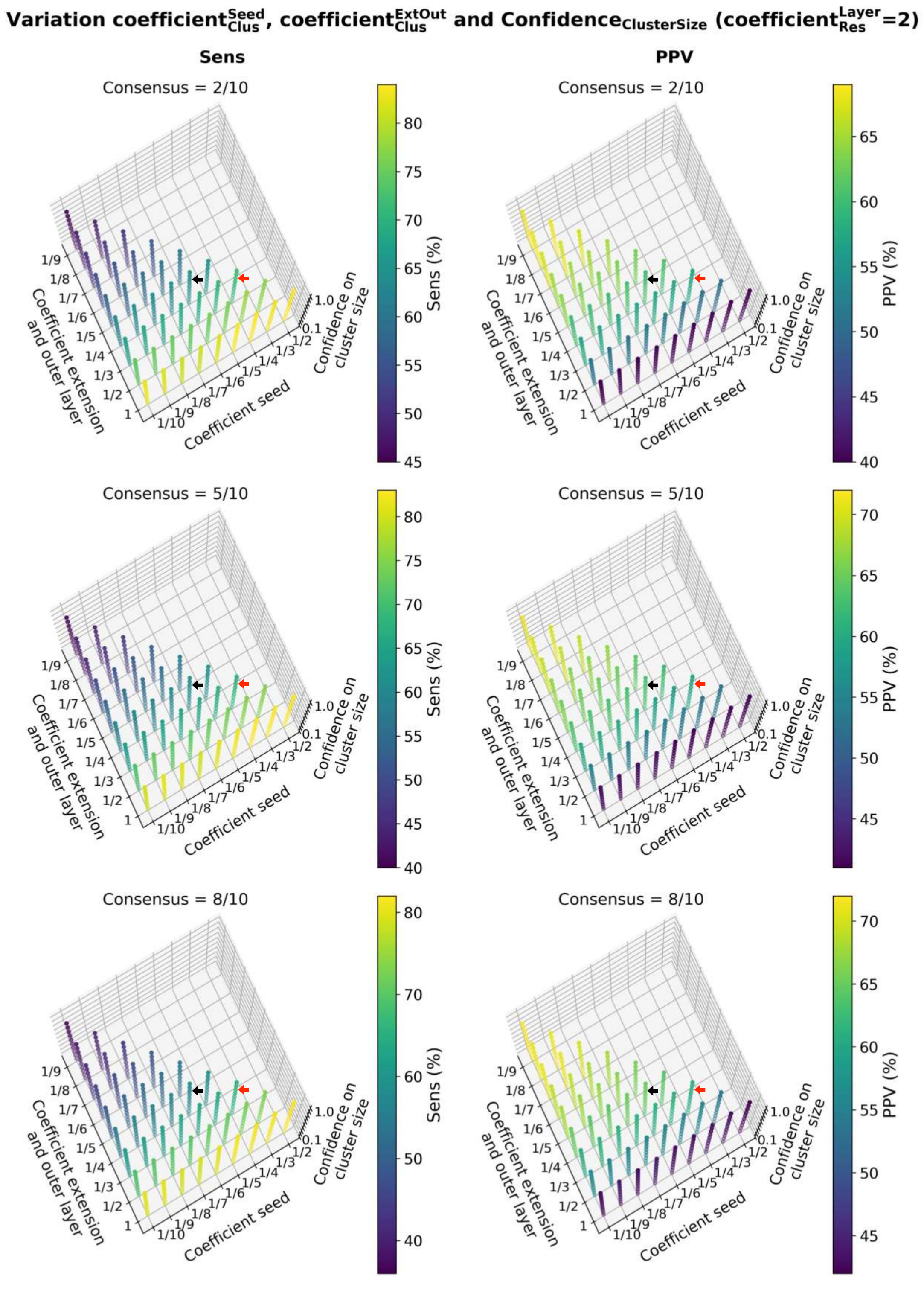

Figure O: **Influence of the three parameters coefficient$^{Seed}_{Clus}$, coefficient$^{ExtOut}_{Clus}$ and confidence$_{clusSize}$ on the sensitivity and PPV values.** coefficient$^{Seed}_{Clus}$ and coefficient$^{ExtOut}_{Clus}$ are multiplied by $f_{intfrac}$ to determine the two cluster thresholds score$^{Seed}_{Clus}$ and score$^{ExtOut}_{Clus}$. Namely, a given seed (resp. extension) is grown until the averaged score of the predicted cluster reaches down score$^{Seed}_{Clus}$ (resp. score$^{ExtOut}_{Clus}$). The third threshold represents the confidence on the cluster size (confidence$_{clusSize}$). Clusters with a size below this confidence level are filtered out. The statistical value is coded by the color. Results obtained from a consensus of 2, 5 and 8 runs out of 10 are reported on top, at the center and bottom, respectively. The calculation was performed on the HR-PDNA187 dataset. Default values chosen in JET$^2_{\mathrm{DNA}}$ for the first stage (not-relaxed values) and the second stage (relaxed values) of prediction are highlighted in black and red, respectively.

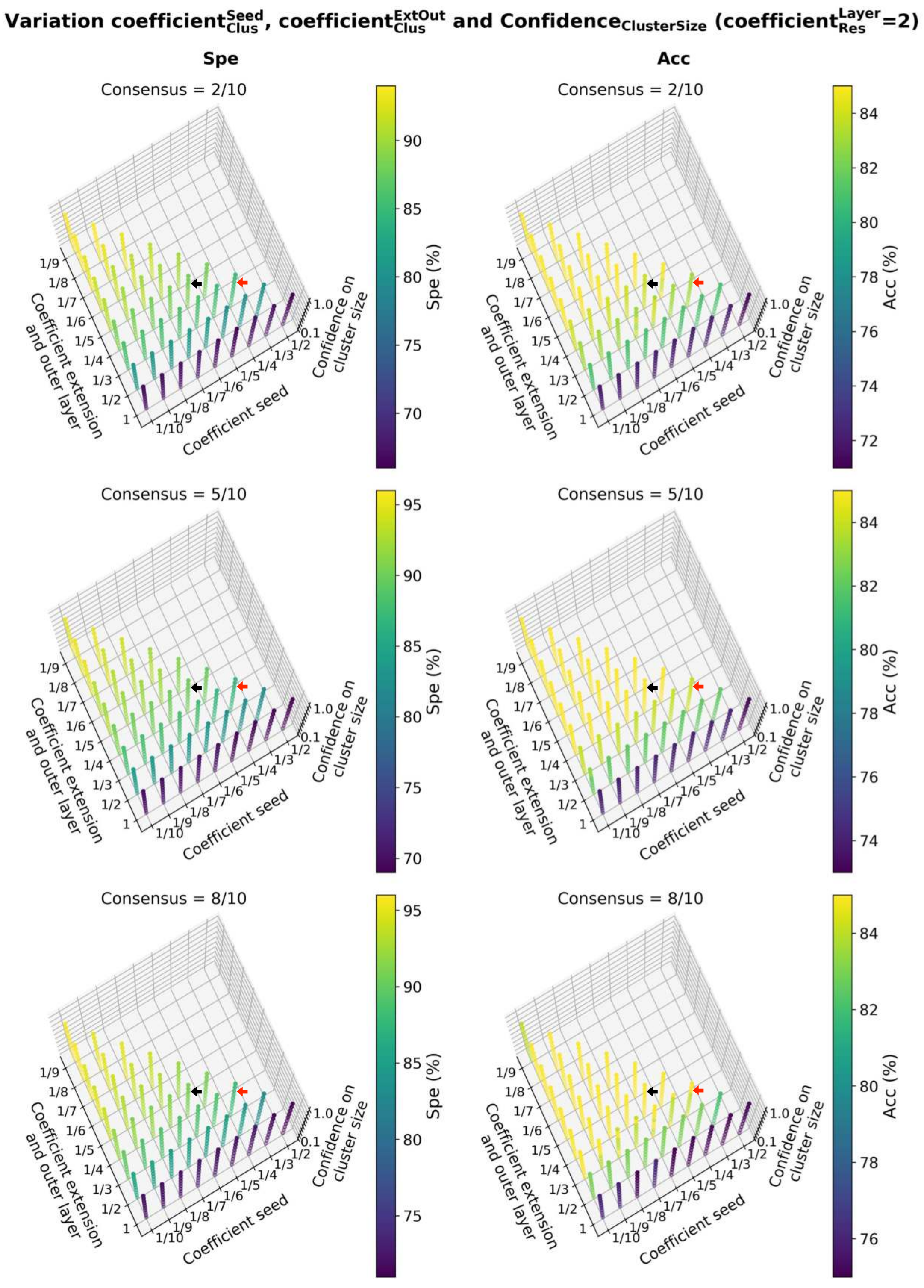

Figure P: **Influence of the three parameters coefficient$^{Seed}_{Clus}$, coefficient$^{ExtOut}_{Clus}$ and confidence$_{clusSize}$ on specificity and accuracy values.** coefficient$^{Seed}_{Clus}$ and coefficient$^{ExtOut}_{Clus}$ are multiplied by $f_{intfrac}$ to determine the two cluster thresholds score$^{Seed}_{Clus}$ and score$^{ExtOut}_{Clus}$. Namely, a given seed (resp. extension) is grown until the averaged score of the predicted cluster reaches down score$^{Seed}_{Clus}$ (resp. score$^{ExtOut}_{Clus}$). The third threshold represents the confidence on the cluster size (confidence$_{clusSize}$). Clusters with a size below this confidence level are filtered out. The statistical value is coded by the color. Results obtained from a consensus of 2, 5 and 8 runs out of 10 are reported on top, at the center and bottom, respectively. The calculation was performed on the HR-PDNA187 dataset. Default values chosen in JET$^2_{\mathrm{DNA}}$ for the first stage (not-relaxed values) and the second stage (relaxed values) of prediction are highlighted in black and red, respectively.

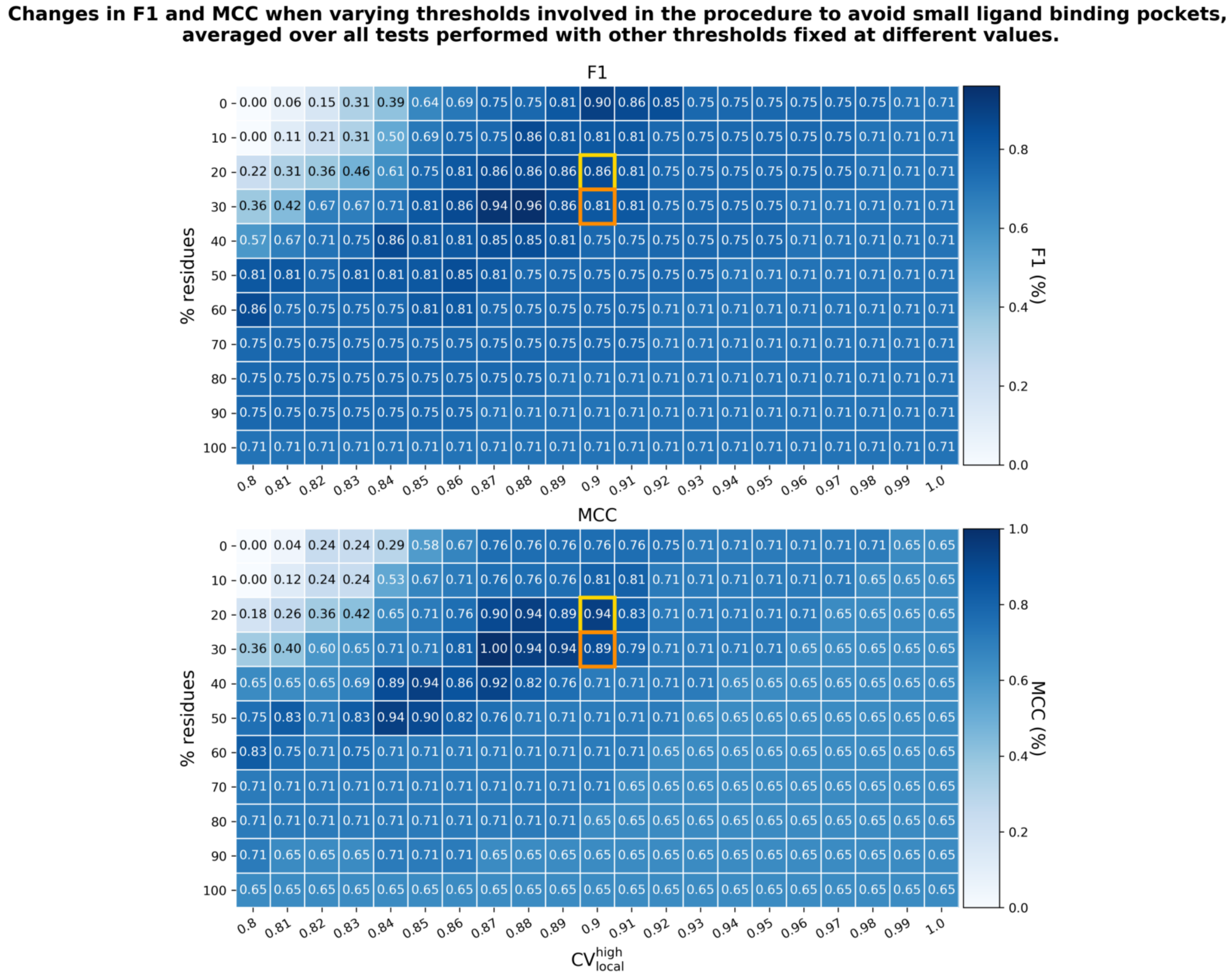

Figure Q: **Influence of the two parameters involved in the procedure to avoid the prediction of small ligand binding pockets on averaged F1 and MCC values.** The $CV_{local}{}^{high}$ (x-axis) and the percentage of residues allowed to show $CV_{local} \geq CV_{local}{}^{high}$ (y-axis) are the two parameters involved in the procedure to avoid the prediction of small ligand binding pockets. In each cell is reported the F1 (on top) or the MCC value (at the bottom) averaged over the six 2D plots in Supp. Fig. 10-11. F1 and MCC values in each 2D plot in Supp. Fig. 10-11 were first normalized with respect to the minimum and maximum values over the plot. The calculation was performed on the HR-PDNA187 dataset. Default values chosen in JET^2_{DNA} for D-SC1 and D-SC2 are highlighted in yellow and orange, respectively.

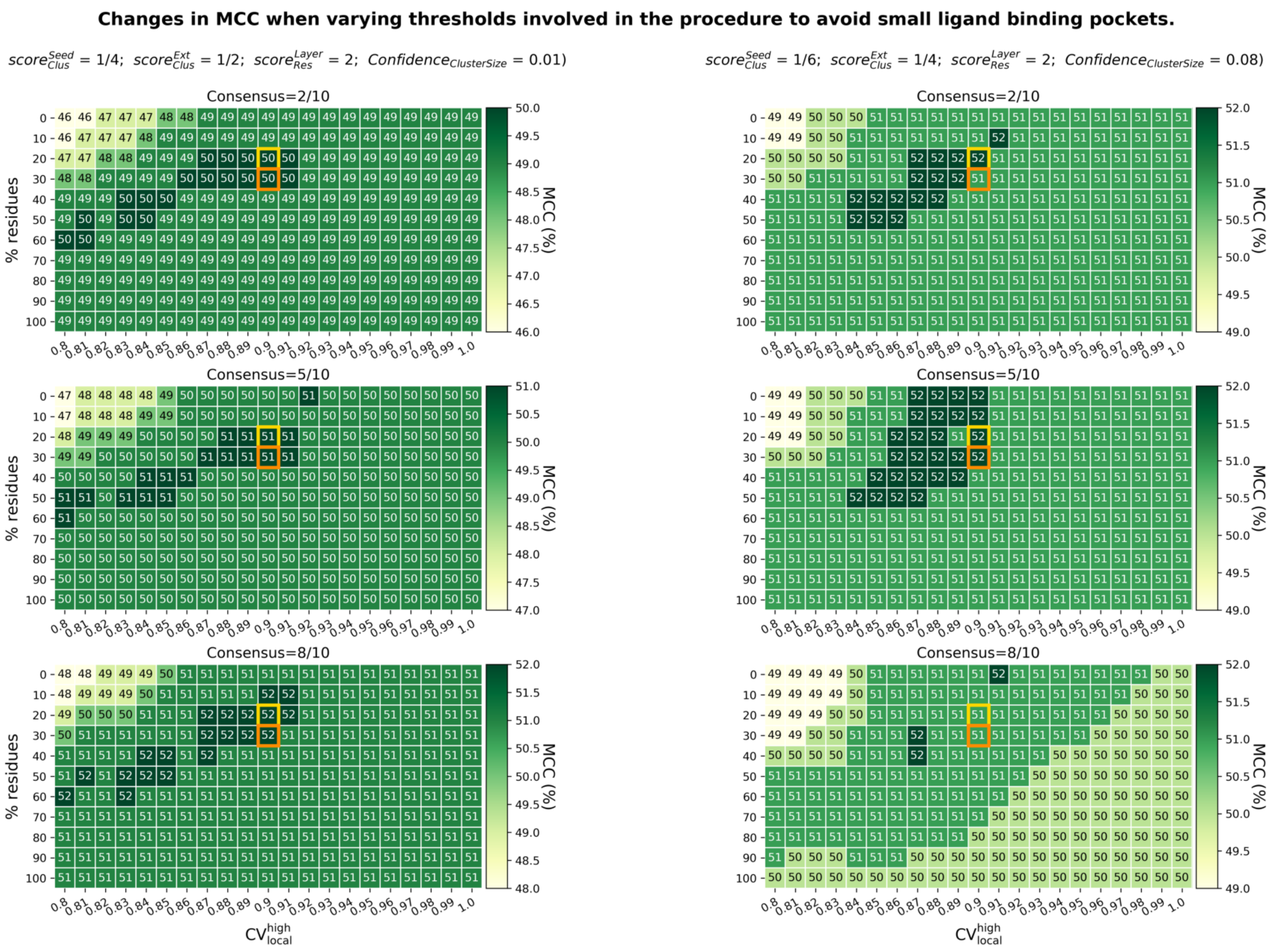

Figure R: **Influence of the two parameters involved in the procedure to avoid the prediction of small ligand binding pockets on MCC values.** The $CV_{local}{}^{high}$ (x-axis) and the percentage of residues allowed to show $CV_{local} \geq CV_{local}{}^{high}$ (y-axis) are the two parameters involved in the procedure to avoid the prediction of small ligand binding pockets. In each cell is reported the averaged MCC value computed on the HR-PDNA187 dataset. Two different sets of $score_{Clus}^{Seed}$, $score_{Clus}^{ExtOut}$, $score_{Res}^{Layer=seed,extension,outer\ layer}$ and $Confidence_{ClusterSize}$ were tried, reported on the left and right columns respectively, to test the stability of results with respect to these other thresholds used in the algorithm. In each column, three 2D plots are reported for predictions obtained with a consensus of 2, 5 and 8 runs out of 10, respectively. Default values chosen in JET^2_{DNA} for D-SC1 and D-SC2 are highlighted in yellow and orange, respectively.

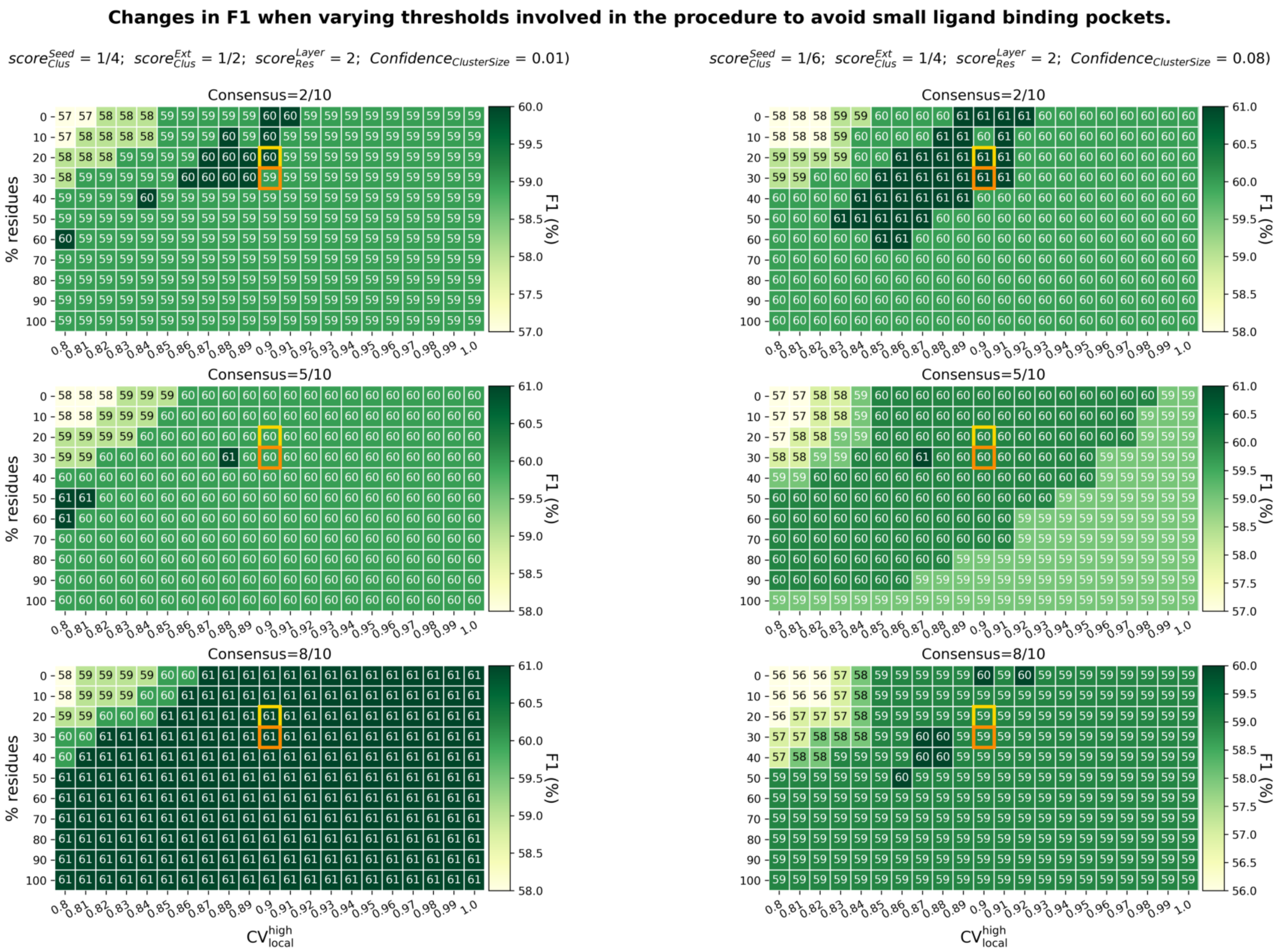

Figure S: **Influence of the two parameters involved in the procedure to avoid the prediction of small ligand binding pockets on F1 values.** The $CV_{local}{}^{high}$ (x-axis) and the percentage of residues allowed to show $CV_{local} \geq CV_{local}{}^{high}$ (y-axis) are the two parameters involved in the procedure to avoid the prediction of small ligand binding pockets. In each cell is reported the averaged F1 value computed on the HR-PDNA187 dataset. Two different sets of $score_{Clus}^{Seed}$, $score_{Clus}^{ExtOut}$, $score_{Res}^{Layer=seed,extension,outer\ layer}$ and $Confidence_{ClusterSize}$ were tried, reported on the left and right columns respectively, to test the stability of results with respect to these other thresholds used in the algorithm. In each column, three 2D plots are reported for predictions obtained with a consensus of 2, 5 and 8 runs out of 10, respectively. Default values chosen in JET^2_{DNA} for D-SC1 and D-SC2 are highlighted in yellow and orange, respectively.

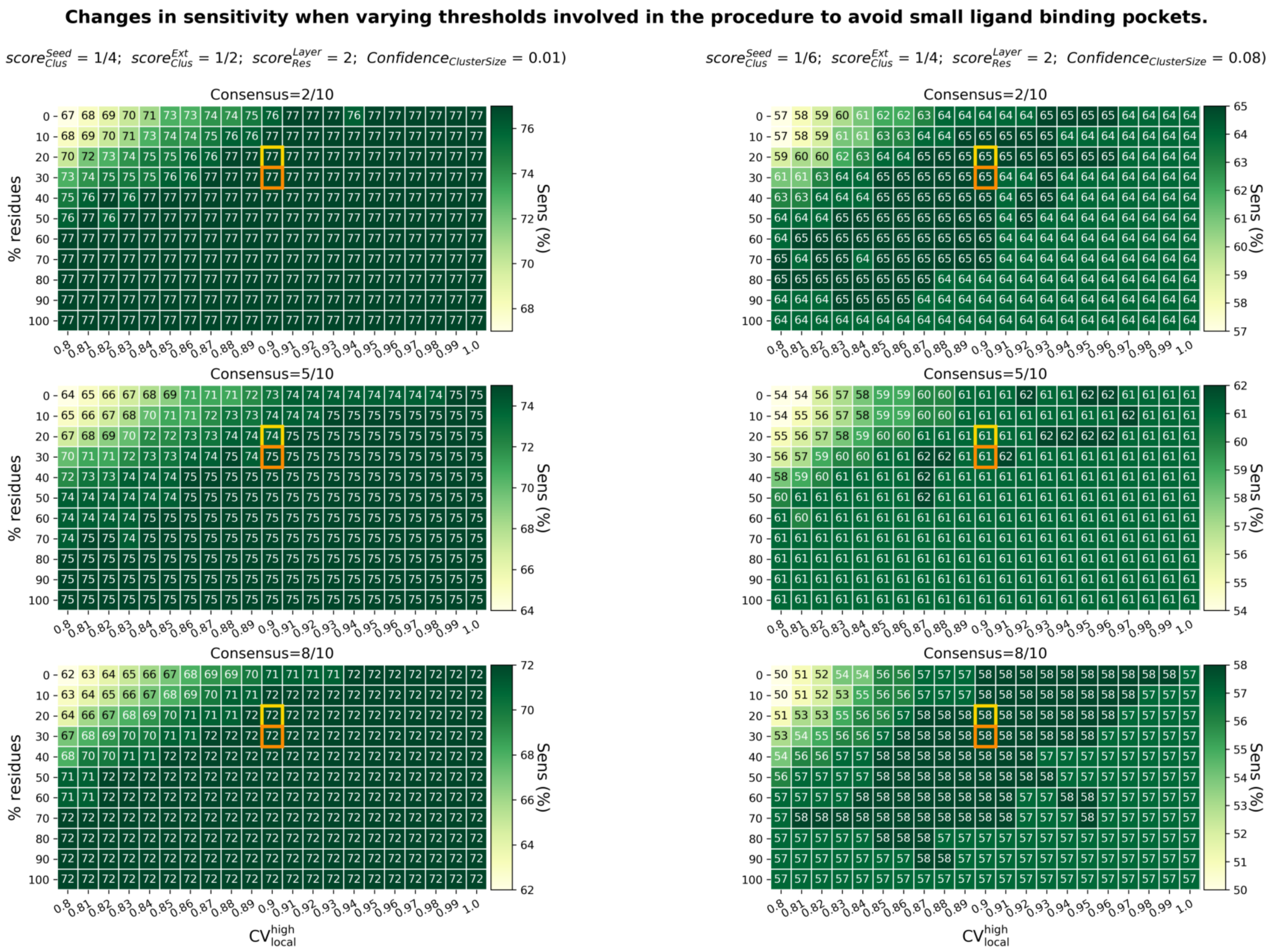

Figure T: **Influence of the two parameters involved in the procedure to avoid the prediction of small ligand binding pockets on sensitivity values.** The $CV_{local}{}^{high}$ (x-axis) and the percentage of residues allowed to show $CV_{local} \geq CV_{local}{}^{high}$ (y-axis) are the two parameters involved in the procedure to avoid the prediction of small ligand binding pockets. In each cell is reported the averaged sensitivity value computed on the HR-PDNA187 dataset. Two different sets of $score^{Seed}_{Clus}$, $score^{ExtOut}_{Clus}$, $score^{Layer=seed,extension,outer\ layer}_{Res}$ and $Confidence_{ClusterSize}$ were tried, reported on the left and right columns respectively, to test the stability of results with respect to these other thresholds used in the algorithm. In each column, three 2D plots are reported for predictions obtained with a consensus of 2, 5 and 8 runs out of 10, respectively. Default values chosen in JET^2_{DNA} for D-SC1 and D-SC2 are highlighted in yellow and orange, respectively.

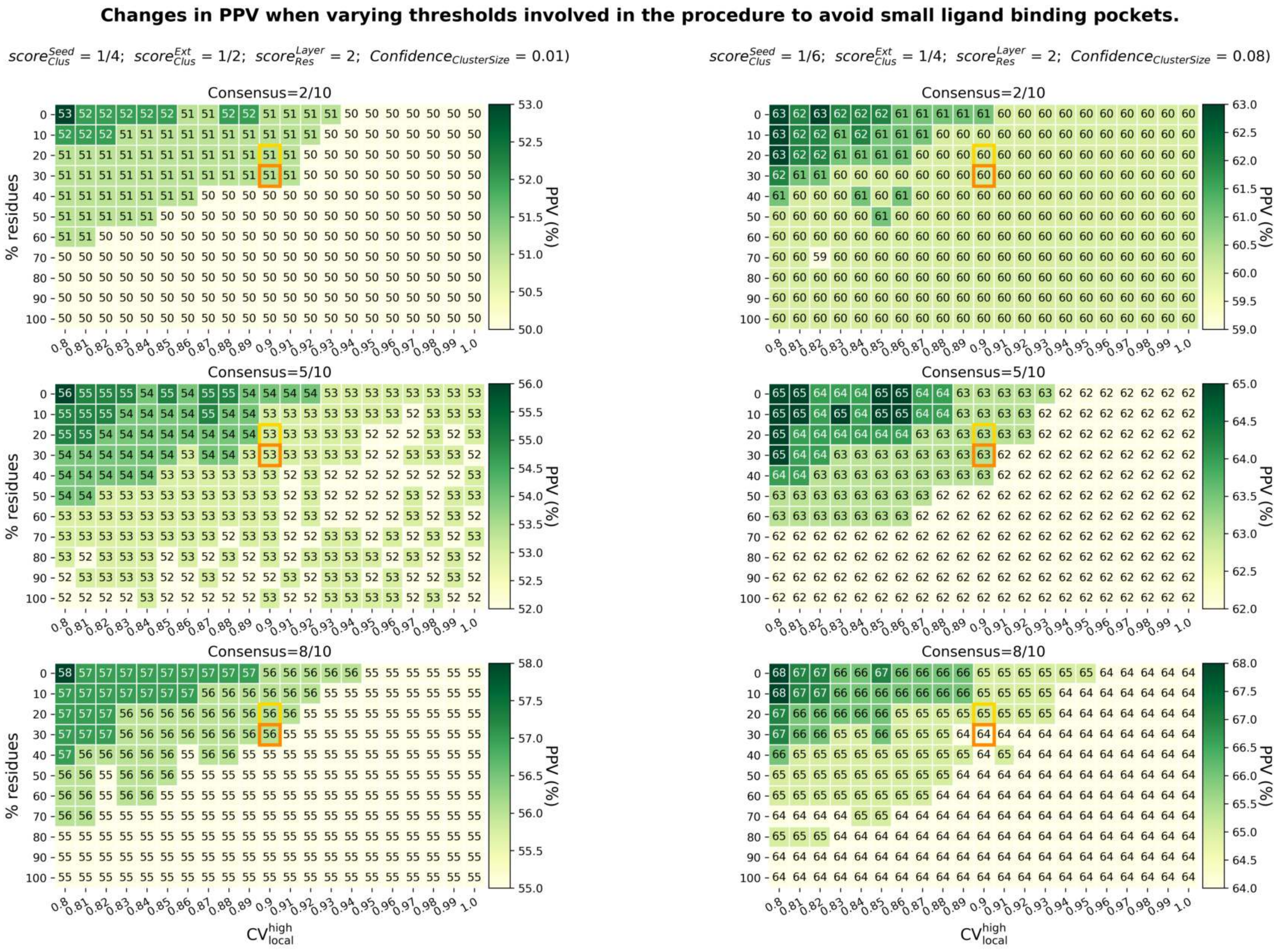

Figure U: **Influence of the two parameters involved in the procedure to avoid the prediction of small ligand binding pockets on PPV values.** The $CV_{local}{}^{high}$ (x-axis) and the percentage of residues allowed to show $CV_{local} \geq CV_{local}{}^{high}$ (y-axis) are the two parameters involved in the procedure to avoid the prediction of small ligand binding pockets. In each cell is reported the averaged PPV value computed on the HR-PDNA187 dataset. Two different sets of $score_{Clus}^{Seed}$, $score_{Clus}^{ExtOut}$, $score_{Res}^{Layer=seed,extension,outer\ layer}$ and $Confidence_{ClusterSize}$ were tried, reported on the left and right columns respectively, to test the stability of results with respect to these other thresholds used in the algorithm. In each column, three 2D plots are reported for predictions obtained with a consensus of 2, 5 and 8 runs out of 10, respectively. Default values chosen in JET^2_{DNA} for D-SC1 and D-SC2 are highlighted in yellow and orange, respectively.

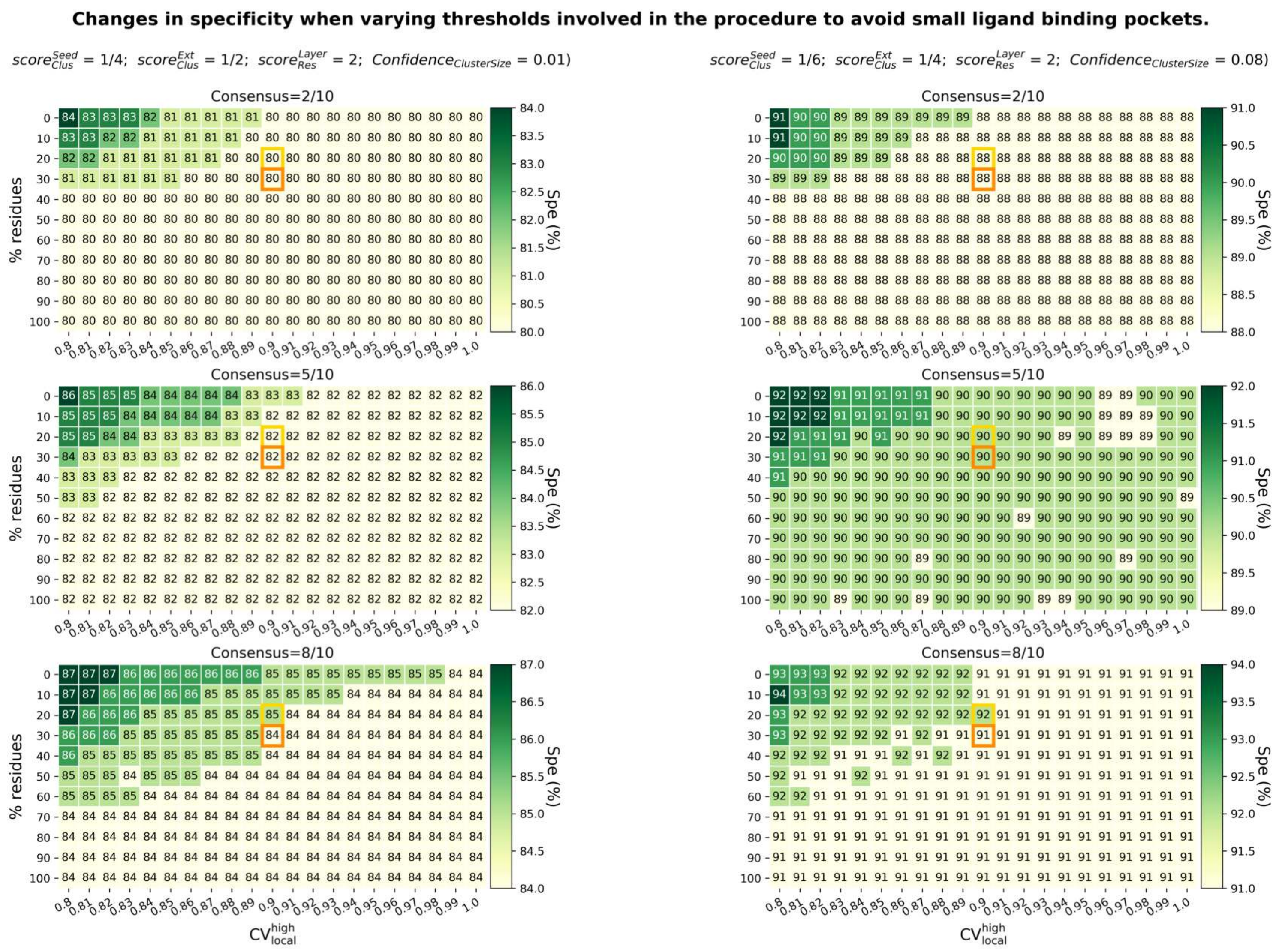

Figure V: **Influence of the two parameters involved in the procedure to avoid the prediction of small ligand binding pockets on specificity values.** The $CV_{local}{}^{high}$ (x-axis) and the percentage of residues allowed to show $CV_{local} \geq CV_{local}{}^{high}$ (y-axis) are the two parameters involved in the procedure to avoid the prediction of small ligand binding pockets. In each cell is reported the averaged specificity value computed on the HR-PDNA187 dataset. Two different sets of $score_{Clus}^{Seed}$, $score_{Clus}^{ExtOut}$, $score_{Res}^{Layer=seed,extension,outer\ layer}$ and $Confidence_{ClusterSize}$ were tried, reported on the left and right columns respectively, to test the stability of results with respect to these other thresholds used in the algorithm. In each column, three 2D plots are reported for predictions obtained with a consensus of 2, 5 and 8 runs out of 10, respectively. Default values chosen in JET^2_{DNA} for D-SC1 and D-SC2 are highlighted in yellow and orange, respectively.

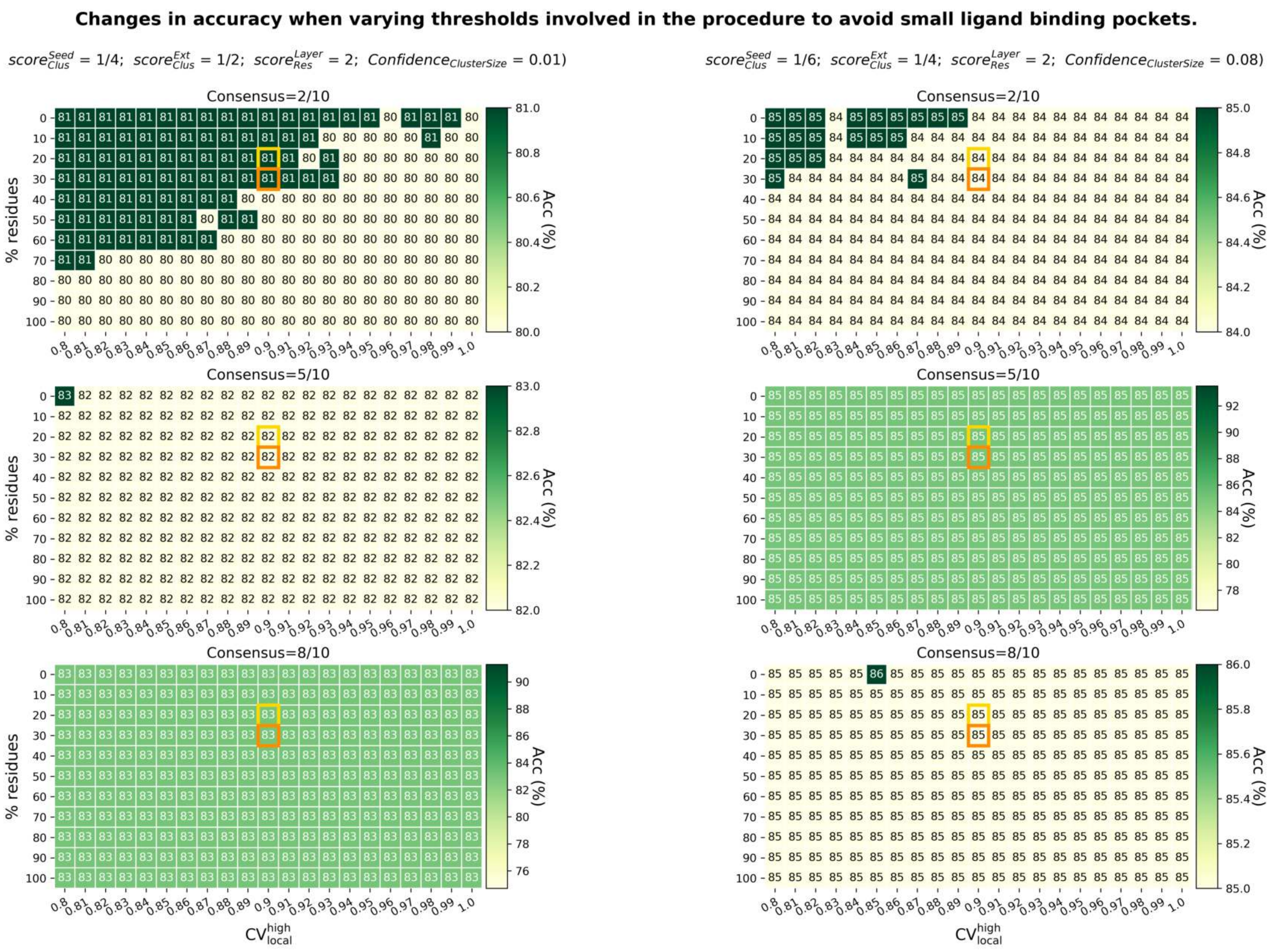

Figure W: **Influence of the two parameters involved in the procedure to avoid the prediction of small ligand binding pockets on accuracy values.** The $CV_{local}{}^{high}$ (x-axis) and the percentage of residues allowed to show $CV_{local} \geq CV_{local}{}^{high}$ (y-axis) are the two parameters involved in the procedure to avoid the prediction of small ligand binding pockets. In each cell is reported the averaged accuracy value computed on the HR-PDNA187 dataset. Two different sets of $score_{Clus}^{Seed}$, $score_{Clus}^{ExtOut}$, $score_{Res}^{Layer=seed,extension,outer\ layer}$ and $Confidence_{ClusterSize}$ were tried, reported on the left and right columns respectively, to test the stability of results with respect to these other thresholds used in the algorithm. In each column, three 2D plots are reported for predictions obtained with a consensus of 2, 5 and 8 runs out of 10, respectively. Default values chosen in JET_{DNA}^2 for D-SC1 and D-SC2 are highlighted in yellow and orange, respectively.
