# Supplementary material for: Multiple protein-DNA interfaces unravelled by evolutionary information, physico-chemical and geometrical properties
Source: PLoS Comput Biol. 2020 Feb 3;16(2):e1007624. doi: 10.1371/journal.pcbi.1007624 (PMC7018136; doi:10.1371/journal.pcbi.1007624)
Supplement: Attachment — Submitted filename: Response_to_reviewers.pdf [file pcbi.1007624.s004.pdf]

Response to reviewers

Reviewer 1

Many different kinds of protein interact with nucleic acids to perform a variety of essential functions (eg enzymes and transcription factors); only a small fraction of the known 3D structures in the Protein Data Bank are of such complexes. This manuscript makes an important contribution to the field. It describes a computational method to identify and predict protein sites that can bind DNA or RNA molecules in a way that makes it possible to interpret the most prominent features underpinning the recognition. The performance of the methods is tested, and compared to other methods, using a carefully composed set of experimentally characterized protein-D/RNA complexes. Somewhat surprisingly the method, which was trained to detect DNA-binding sites, does quite well also regarding RNA-binding sites.

I have only a couple of minor items that the authors should look at:

The six performance measures are only presented as mathematical expressions (page 8) - to help the reader it would be useful to also explain what they measure, and how they relate to each other.

We added the explanation of the six performance measures and how they relate to each other in section "Evaluation of performances".

What is on the y-axis in Fig 4 (% of what?)?

On the y-axis in Fig. 4 are reported the statistical performance values (Sens, PPV, Spe, Acc, F1, MCC) given in percentages. Specifically, we multiplied the original values by 100, in order to obtain percentages in the range [-100, 100] for MCC values, and [0, 100] for the other statistical measures. To clarify this point, we added a few lines in section "Evaluation of performances" explaining more clearly how we computed percentages of statistical values reported in all figures and tables. Moreover, we added a clarifying sentence in Fig. 3 and Fig. 4, and we modified the y-axis label of Fig. 4 in order to make it more explicit.

Reference 32 is incomplete.

We corrected reference number [32] in the previous version of the manuscript, now number [33].

Reviewer 2

The manuscript builds upon the already published papers by this group on protein-protein interaction to decipher the complex protein surface that is used for recognition of DNA. The complications in the process are in plenty, such as the conformational change during binding, the use of the same protein surface region for binding several partners, or sometimes a RNA molecule. The authors have developed a method, JET^2_{DNA} to predict protein-DNA interfaces and characterize their properties. The method has been compared to several machine-learning methods and found to be robust. During the process the authors have developed a new dataset of 187 protein DNA complex structures, along with a subset of 82 associated unbound structures, and this could serve as a reference benchmark for prediction for DNA-binding site prediction and docking studies. Overall the study has been done with rigor, though it is quite daunting to comprehend the large volume of data provided in the supplementary material. The text cites some supporting tables. But the supporting file that I could download has only figures.

We are very sorry about that. We just realized that the supplementary tables were missing from the files uploaded during the first submission. We uploaded them in this second submission.

The authors may look into the following minor corrections.

1) Pg 2, line 34 mentions of support-core-rim model, citing reference [33]. I think it is useful to also include the original paper dealing with core-rim model (Chakrabarti and Janin, Proteins (2002), 47, 334). In fact, protein-DNA interfaces were also dissection in the similar fashion and correlated with the degree of conservation by the same workers , reference [54].

We added a sentence in the introduction to mention the original papers introducing the core-rim model for dissecting protein-protein (references [55,56]) and protein-DNA interfaces (references [54]).

2) Pg 3, line 43. 'independent test set of 82 nucleic acid binding proteins'. Later in Pg 4, line 107, (TEST-DBP24) is mentioned. I was wondering why these 24 proteins got excluded from the HR-PDNA187 dataset curated by the authors?

We added Supp. Table S2 where we list the 24 complexes present in TEST-DBP24 and the reasons they were excluded from HR-PDNA187, despite their good (< 2.5 Å) resolution.

Specifically:

- 3UGO, 4FPV, 4GZ0, 4HIO, 4HQB, 4JRP, 4LJR, 4LUP, 4OWX: discarded in our dataset because single stranded DNA (page 3, line 77).

- 3VOK, 3ZDB, 4E0J, 4HLY, 4HQX: discarded in our dataset because the asymmetric unit does not contain a complete biological unit (page 3, line 76).

- 3ZH2, 4BXO, 4LMG, 4NDH, 4NI7, 4NM6: not present in our original list downloaded from NDB (Nucleic Acids Database).

- 3VD6: homologous to 4HC9 and thus discarded in our dataset by PISCES; then 4HC9 was itself manually discarded in our dataset because interfaces of asymmetric and biological units did not match (page 3, lines 80-81).

- 3U3W, 4HP1, 4L0Y: discarded in our dataset by PISCES because having a sequence identity in the range 25-30% compared to 3S8Q, 3QMD, 1YO5, respectively.

3) Pg 5, Circular variance is defined based on neighboring points. What are these points, individual atoms, or C-alpha atoms, or atoms, excluding those belonging to the same residue? As mentioned in the manuscript, a value close to 0 is very unlikely, especially if the radius is 12 Å. Has the justification for using the values of 12 and 100 Å provided? The latter value seems to be too large for a standard-size protein. Some explanations are needed about the typical values of the two CV parameters, and why (1-CV) values are used in Fig. 1(b). Line 227, and explanation is needed why a value of CVglobal > 0.6 indicates a very concave region.

We modified the "Circular variance" paragraph to clarify its definition, calculation and the definition of its threshold values.
The CV value of an atom i is computed as:

$$CV(i) = 1 - \frac{1}{n_i} \left| \sum_{j \neq i, r_i \leq r_c} \frac{\vec{r_{ij}}}{||\vec{r_{ij}}||} \right| \quad (1)$$

where n_i is the number of all individual atoms distant by less than r_c Å from atom i. The CV value of a residue is then computed as the average of the atomic CVs, over all its atoms. The values of r_c for CV_{local} (r_c = 12Å) and $\text{CV}_{\text{global}}$ (r_c = 100Å) are the same ones we already used in [32] to predict protein-protein interfaces. In [32], we showed that thresholds in the range 10-14Å for CV_{local} would not change the results. Concerning the threshold value fixed for $\text{CV}_{\text{global}}$, a value of 100Åensures the inclusion of essentially all protein atoms in the calculation. If the protein is smaller than 100Å, then the $\text{CV}_{\text{global}}$ values will not be affected by the fact that the threshold is bigger.

Given a residue, its CV value reflects the protein density around it. Thus, a low CV value (close to 0) indicates that a residue is located in a protruding region of the protein surface, while a high value (close to 1) indicates it is buried within the protein. By consequence, the complement measure (1- CV) show low values for buried residues and high values for protruding ones. Since we want to assign high score to residues of interest, we use the CV_{global} measure to assign high values to globally buried residues, and (1-CV_{local}) to assign high values to locally protruding ones. In Fig. 1(b), we used (1 - CV_{local}) in the formulas of D-SC1 and D-SC3 since we want to assign high scores to locally protruding residues when using these two scoring schemes, thus the ones having high (1 - CV_{local}) values. We explain this in paragraph "Three strategies to detect protein-DNA interfaces".
We added an additional supplementary figure (Fig. S5) to make clearer why a $CV_{global} > 0.6$ is used to indicate very concave regions. In this figure, we report five examples: in the first three there is an overlap between the concave regions and the respective DNA-binding sites (Supp. Fig. S5(a), on top), while in the other two the concave regions are distant from the respective DNA-binding sites (Supp. Fig. S5(b), on top). In all the five cases, a threshold of $CV_{global} > 0.6$ well defines globally concave protein regions, giving a good compromise between too small and too large ones. In the cases where the protein concave regions coincide with the respective DNA-binding sites, the JET^2_{DNA} automated clustering procedure was able to correctly choose, for the first and third cases, the D-SC2 scoring scheme designed to accurately predict these types of protein-DNA interfaces (Fig. S5, a, on bottom). In the other two cases where the concave regions are distant from the respective DNA-binding sites, the JET^2_{DNA} automated clustering procedure is able to avoid them and predict the correct DNA-binding sites by choosing D-SC1 (Fig. S5, a, on bottom), likely because the conservation and physico-chemical properties of the concave regions do not match with the ones expected for DNA-binding sites.

4) Fig. 1(a). Whatever is written in yellow is not legible. Likewise, in Figure S2.
We modified Fig.1(a) and Figure S2 in order to make legible the parts in yellow.

5) Lines 279-285. More clarity is needed in the definition of core, rim and support residues. Ref [33] used rASA values in the free and complex states to define the residues. The way it is written, support residues are buried ('both'?) in presence and absence of DNA - but that the degree of burial changes is not apparent. Similarly, the degree of exposures for rim residues? Lines 290-291. If there is any reference to support the statement that the core residues fit into grooves, whereas the support interacts with backbone, this needs to be provided.

The exact percentage of rASA of support, core and rim residues and their degree of burial changes are reported in *Materials and Methods*, section "Definition of interface residues", where we firstly define them. Specifically, interface residues were defined as those being more buried in the presence of DNA than in the free form ($\Delta rasa > 0$). We divide interface residues in: support residues that are buried both in presence ($rasa_{DNA} < 25\%$) and absence of DNA ($rasa_{free} < 25\%$); core residues that are exposed in absence of DNA ($rasa_{free} \geq 25\%$) and become buried upon binding ($rasa_{DNA} < 25\%$); rim residues that are exposed in presence

($rasa_{DNA} \geq 25\%$) and in absence ($rasa_{free} \geq 25\%$) of DNA.
To support the statement that the core residues fit into grooves, whereas the support interacts with backbone, we performed some additional analysis, added in Fig.2a-d. We computed: (a) the total number of atomic contacts between protein interface residues, divided in support, core and rim, and DNA backbone (plain color) and DNA base atoms (diagonal hatching); (b) the distribution of the proportion of residues comprised in support, core and rim; (c) the distributions of the average number of atomic contacts (defined by an atomic distance <5Å) per interface residue in support, core and rim; (d) the distributions of the proportion of atomic contacts established by support, core and rim residues with DNA backbone and DNA base atoms. The results of these additional analysis demonstrated that overall *core* residues establish the majority of the atomic contacts (defined by an atomic distance <5 Å) (Fig. 2a and c) with both DNA backbone and DNA base atoms (Fig. 2a), despite a comparable number of support, core and rim residues in HR-PDNA187 (Fig. 2b) (support: 2501, core: 2958, rim: 2891). More than 75% of protein-DNA base contacts involve *core* residues (Fig. 2a and d), while *support* and *rim* residues prevalently establish contacts with DNA backbone atoms (Fig. 2a and d). We added these additional considerations in paragraph "*Support-Core-Rim* vs *Core-Support-Rim* model for protein-DNA interactions"

6) Lines 300-301. "DNA binding sites ... are more conserved that the rest of the protein". The "rest" includes the protein interior, or just the rest of the surface? Where are the data for the "rest"?

The "rest" includes the protein interior. We reformulated caption of Fig. 2 and also the text in paragraph "Characteristics of protein-DNA interfaces" to mention that the percentages are calculated taking into account the entire protein and not only the surface.
We did not explicitly reported values for non-interacting residues, because the median value, with respect to which we compare interacting ones, is already computed considering the entire protein, thus taking into account also the "rest" of the protein.
