# Supplementary material for: Multiple protein-DNA interfaces unravelled by evolutionary information, physico-chemical and geometrical properties
Source: PLoS Comput Biol. 2020 Feb 3;16(2):e1007624. doi: 10.1371/journal.pcbi.1007624 (PMC7018136; doi:10.1371/journal.pcbi.1007624)
Supplement: Attachment — Submitted filename: PLoS-CB-comments.docx [file pcbi.1007624.s003.docx]

The manuscript builds upon the already published papers by this group on protein-protein interaction to decipher the complex protein surface that is used for recognition of DNA. The complications in the process are in plenty, such as the conformational change during binding, the use of the same protein surface region for binding several partners, or sometimes a RNA molecule. The authors have developed a method, JET^2^*_DNA_* to predict protein-DNA interfaces and characterize their properties. The method has been compared to several machine-learning methods and found to be robust. During the process the authors have developed a new dataset of 187 protein-DNA complex structures, along with a subset of 82 associated unbound structures, and this could serve as a reference benchmark for prediction for DNA-binding site prediction and docking studies

Overall the study has been done with rigor, though it is quite daunting to comprehend the large volume of data provided in the supplementary material.

The text cites some supporting tables. But the supporting file that I could download has only figures.

The authors may look into the following minor corrections.

1) Pg 2, line 34 mentions of support-core-rim model, citing reference [33]. I think it is useful to also include the original paper dealing with core-rim model (Chakrabarti and Janin, Proteins (2002), 47, 334). In fact, protein-DNA interfaces were also dissection in the similar fashion and correlated with the degree of conservation by the same workers , reference [54].

2) Pg 3, line 43. ‘independent test set of 82 nucleic acid binding proteins’. Later in Pg 4, line 107, (TEST-DBP24) is mentioned. I was wondering why these 24 proteins got excluded from the HR-PDNA187 dataset curated by the authors?

3) Pg 5, Circular variance is defined based on neighboring points. What are these points, individual atoms, or C-alpha atoms, or atoms, excluding those belonging to the same residue? As mentioned in the manuscript, a value close to 0 is very unlikely, especially if the radius is 12 Å. Has the justification for using the values of 12 and 100 Å provided? The latter value seems to be too large for a standard-size protein. Some explanations are needed about the typical values of the two CV parameters, and why (1-CV) values are used in Fig. 1(b). Line 227, and explanation is needed why a value of CV_global_ > 0.6 indicates a very concave region.

4) Fig. 1(a). Whatever is written in yellow is not legible. Likewise, in Figure S2.

5) Lines 279-285. More clarity is needed in the definition of core, rim and support residues. Ref [33] used rASA values in the free and complex states to define the residues. The way it is written, support residues are buried (‘both’?) in presence and absence of DNA – but that the degree of burial changes is not apparent. Similarly, the degree of exposures for rim residues? Lines 290-291. If there is any reference to support the statement that the core residues fit into grooves, whereas the support interacts with backbone, this needs to be provided.

6) Lines 300-301. “DNA binding sites … are more conserved that the rest of the protein”. The “rest” includes the protein interior, or just the rest of the surface? Where are the data for the “rest”?
